# Finite-Time Thermodynamics and Complex Energy Landscapes: A Perspective

**DOI:** 10.3390/e27080819

**Published:** 2025-08-01

**Authors:** Johann Christian Schön

**Affiliations:** Max-Planck-Institute for Solid State Research, Heisenbergstr. 1, D-70569 Stuttgart, Germany; c.schoen@fkf.mpg.de

**Keywords:** finite-time thermodynamics, energy landscapes, thermodynamic processes, phase transitions, glasses, optimal control, metastable phases, free energy calculations

## Abstract

Finite-time thermodynamics (FTT) describes the study of thermodynamic processes that take place in finite time. Due to the finite-time requirement, in general the system cannot move from equilibrium state to equilibrium state. As a consequence, excess entropy is generated, available work is reduced, and/or the maximally achievable efficiency is not achieved; minimizing these negative side-effects constitutes an optimal control problem. Particularly challenging are processes and cycles that involve phase transitions of the working fluid material or the target material of a synthesis process, especially since most materials reside on a highly complex energy landscape exhibiting alternative metastable phases or glassy states. In this perspective, we discuss the issues and challenges involved in dealing with such materials when performing thermodynamic processes that include phase transitions in finite time. We focus on thermodynamic cycles with one back-and-forth transition and the generation of new materials via a phase transition; other systems discussed concern the computation of free energy differences and the general applicability of FTT to systems outside the realm of chemistry and physics that exhibit cost function landscapes with phase transition-like dynamics.

## 1. Introduction

One of the fundamental building blocks of physics characterized by its own set of basic laws and equations is the field of thermodynamics [1,2,3]. Its three laws are well-known: the conservation of energy in an isolated system, the non-decrease of the entropy of an isolated system, and the fact that the entropy becomes constant at zero temperature. Sometimes a zeroth law is added, i.e., that two systems that each are in equilibrium with a third (reference) system are also in equilibrium with each other. A beautiful body of rules and laws of thermodynamics has been built on this foundation, guiding the development of modern technology wherever the application and exchange of work and heat are involved.

In parallel, the field of mechanics [4] has evolved from early formulations of force laws via Newton’s equations and their derivation from optimality principles for systems described by few parameters to the study of multi-particle systems [5]. Such systems appear in the form of rigid, deformable, or fluid bodies that can be described on some level of resolution in terms of continuum physics [6], in particular by the theory of elasticity and fluid dynamics. Going beyond the fundamental entity of continuum physics, the so-called material particle or material point [6]—characterized and named by its evolving position in space on the mesoscopic level—, it is possible to address even smaller length scales, in particular the atomic level. On this level of the ladder of description, the number of (to a large extent indistinguishable) particles which a macroscopic system consists of usually exceeds the capacity to compute the evolution of the system according to the laws of mechanics (as well as electromagnetism and quantum mechanics). Even a precise definition or measurement of the initial state of the system at a given moment in time cannot be established with the accuracy necessary to avoid getting caught up in the whirlwind of chaotic behavior of such systems on the microscopic scale. Furthermore, for the vast majority of multi-atom systems, the potential energy function over the state space of classical atom configurations, or more generally quantum mechanical eigenstates, exhibits a highly complex multi-minima structure, thereby introducing a multitude of time scales relevant for characterization of the system’s dynamics [7,8].

Nevertheless, we can formulate empirical laws that can predict the behavior of such macroscopic systems at the macroscopic, and to a certain extent the mesoscopic level, in experiment, indicating that some kind of order on the meso- and macroscopic level can emerge from the underlying chaos of the multi-atom dynamics. The bridge between the atom-level description and that on the level of continuum mechanics and macroscopic thermodynamics is the field of statistical mechanics [9]. Underlying statistical mechanics is the assumption of equal probability for every microstate *i* of an isolated system in the microcanonical ensemble, which allows us to compute a straightforward ensemble average for any observable $O_{\alpha }$. By interacting with the environment, we can introduce the intensive thermodynamic variables as Lagrange multipliers into the ensemble description (e.g., temperature as the control for the exchange of energy with the environment), while the assumption of ergodicity, i.e., the equality of the time average along infinitely long trajectories and the ensemble average over all microstates for all observables, establishes the connection to the classical or quantum mechanical time evolution of the system on the atomic level.

However, in reality we are dealing with finite times, both regarding our measurements of observables and the thermodynamic processes of interest, with potentially enormous consequences. Here, we now meet the field of finite-time thermodynamics (FTT) [10,11,12,13]. This field was founded about 50 years ago [14,15], with earlier discussions going back nearly 100 years [16,17]; it has since blossomed and found many applications within both physics and chemistry as well as in engineering [18] and economics [10,19].

Below, after some preliminary remarks, we explore the issue of finite-time thermodynamics in the context of thermodynamically metastable systems in more detail. While the issue of establishing ergodicity and the stochastic or deterministic modeling of the evolution of a material on the atomic level is of great interest by itself and involves the complex energy landscape on its most elementary level, we will approach this analysis from the thermodynamic side by adding mesoscopic and atom-level aspects on a step-by-step basis. As the paradigmatic class of systems, we will consider the chemical materials that serve as the material substrate of the engines that execute the processes in thermodynamic space that are analyzed in the context of finite-time thermodynamics.

## 2. Preliminaries

### 2.1. Transition from Mechanics to Thermodynamics via Statistical Mechanics

Representing a very simple but immensely powerful assumption, the equal likelihood postulate states that “For an isolated system in equilibrium within itself with a given total energy *E*, volume *V* and number of particles *N*, the likelihood of the system to occupy one of the *M* feasible microstates with energy *E* is the same for each microstate, i.e., 1/M”. This assumption makes it possible to show or at least justify that the laws of thermodynamics should apply to such a macroscopic system [3,9]. The starting point is the definition of an entropy $S\left(E,V,N\right)=k_{B}\ln M$. In the next step, thermodynamic intensities $I_{A}$ such as temperature *T* or pressure *p* can be introduced by allowing exchange of extensive quantities *A*, such as energy or volume with the environment. The state of the combined system we observe is now the one where the extensive quantity *A* is split between the original system and the environment ($A=A_{orig}+A_{env}$) in the most likely way; that is, among the many possibilities to achieve this split, we observe the one with the maximum number of microstates. Determining this split for a given system involves the side condition $A=A_{orig}+A_{env}$; the Lagrange multiplier that takes this into account corresponds precisely to the intensity $I_{A}$ associated with the extensive quantity *A*, such that the original system and the environment are in equilibrium with each other if the intensity $I_{A}$ is the same for both systems: $I_{A_{orig}}=I_{A_{env}}$. For example, the temperature follows from the exchange of energy, while the pressure follows from the exchange of volume.

If we let the system interact with a generic environment at a temperature *T* via exchange of energy, the probability of each microstate *i* is then weighted by the well-known Boltzmann-factor $p_{i}=\exp\left(-E_{i}/T\right)/\sum_{j}\exp\left(-E_{j}/T\right)$, where the sum in the denominator extends over all microstates of the system. As a consequence, we compute the expected properties $O_{\alpha }\left(\mathrm{system}\right)$ of the system in equilibrium via the average of the properties $O_{\alpha }\left(i\right)$ for each microstate *i* over the ensemble of microstates {i} weighted by their likelihood $p_{i}$: $O_{\alpha }\left(\mathrm{system}\right)={\langle O_{\alpha }\rangle }_{ens}=\sum_{i}O_{\alpha }\left(i\right)p_{i}$. Because these properties will usually be different for each microstate, in our measurement we need to sample a large enough set of microstates that is representative of the whole system in order to determine $O_{\alpha }\left(\mathrm{system}\right)$ with the desired accuracy.

Such a measurement in an experiment constitutes a time average $\left(1/t_{obs}\right)\int_{0}^{t_{obs}}O_{\alpha }\left(i\left(t\right)\right)dt$$={\langle O_{\alpha }\rangle }_{t_{obs}}$ along the trajectory in state space i(t) over the observation time $t_{obs}$. Unless the system has some unusual symmetry properties, the difference between the ensemble and the time average ($|{\langle O_{\alpha }\rangle }_{ens}-{\langle O_{\alpha }\rangle }_{t_{obs}}|=a\left(O_{\alpha };t_{obs}\right)$) is usually not zero for finite observation times $t_{obs}$, and one might even have to go to some effort to prove that it is zero in the limit of infinite observation times. However, we can obtain a quantitative handle on this problem by defining a system to be in (thermodynamic) equilibrium on time scale $t_{obs}$, if $a\left(O_{\alpha };t_{obs}\right)<a_{eq}\left(O_{\alpha }\right)$ for a defined level of accuracy $a_{eq}\left(O_{\alpha }\right)$ for all observation times larger than $t_{obs}$ [20]. In fact, this allows us to define an equilibration time $\tau_{eq}$ as the smallest such observation time. Of course, this can only be a probabilistic definition; we must consider averages of the observation times $t_{obs}$ at which the value of $a(O_{\alpha };t_{obs})$ falls below the critical value $a_{eq}\left(O_{\alpha }\right)$ for a given trajectory, where the average is taken over all trajectories of the evolving system starting from all possible microstates of the system when defining $\tau_{eq}$ (and similarly regarding the definition of the escape time below) [21]. Due to the importance of the agreement between time and ensemble averages in the practical definition of thermodynamic equilibrium, we say that the system is ergodic for the observable $O_{\alpha }$ on time scales longer than $\tau_{eq}\left(O_{\alpha }\right)$ with accuracy $a_{eq}\left(O_{\alpha }\right)$ if the difference between time and ensemble averages is smaller than this accuracy. In practice, one would prefer $a(O_{\alpha };t_{obs})$ to be dimensionless normalized quantities; we can achieve this by dividing $|{\langle O_{\alpha }\rangle }_{ens}-{\langle O_{\alpha }\rangle }_{t_{obs}}|$ by $|{\langle O_{\alpha }\rangle }_{ens}|$, i.e., the ergodicity criterion would be that $a^{norm}\left(O_{\alpha };t_{obs}\right)=|{\langle O_{\alpha }\rangle }_{ens}-{\langle O_{\alpha }\rangle }_{t_{obs}}\left|/\right|{\langle O_{\alpha }\rangle }_{ens}|$ be smaller than $a_{eq}\left(O_{\alpha }\right)$. However, for the purpose of this presentation, we will ignore this more technical aspect.

We note that this definition of equilibrium and ergodicity only applies to the observable $O_{\alpha }$, while our common-sense definition of a system being in thermodynamic equilibrium assumes that for all observables of interest the difference between ensemble and time averages is below the required level of accuracy. Furthermore, in principle all of these variables $O_{\alpha },O_{\beta },\ldots$ could have different prescribed levels of accuracy, i.e., $a_{eq}\left(O_{\alpha }\right)\neq a_{eq}\left(O_{\beta }\right)$. In particular, this also implies that $\tau_{eq}$ is only defined for a given observable or set of observables, i.e., in general $\tau_{eq}\left(O_{\alpha }\right)\neq \tau_{eq}\left(O_{\beta }\right)$. In practice, we would focus on a finite set of observables which are most relevant for our description of the system and define the equilibration time $\tau_{eq}$ as the largest among the set of observable dependent equilibration times $\tau_{eq}\left(O_{\alpha }\right)$. In principle, we can achieve such a “complete equilibrium” if we employ the *M* statistical likelihoods $L_{i}$ of all *M* microstates *i* as the observables which must fulfill the ergodicity requirement; since in the ensemble picture ${\langle L_{i}\rangle }_{ens}=p_{i}$, the requirement would be $|{\langle L_{i}\rangle }_{t_{obs}}-p_{i}|<a_{eq}\left(L_{i}\right)$ for all microstates *i*. Clearly, if all microstates of a system are visited according to their ensemble equilibrium probability, then the time averages of all their property observables must also fulfill the ergodicity requirement.

The condition of ergodicity is usually non-trivial to prove for a given physical system, at least if one wants to prove that we can make $a_{eq}\left(O_{\alpha }\right)$ arbitrarily small in the limit $t_{obs}\to \infty$. There has been considerable discussion of this issue in the literature [3,22,23,24], since it is often unclear whether it is possible to reach (or be arbitrarily close to) every other microstate from every initial microstate of the system. For our purposes, we will always assume that $a_{eq}\left(O_{\alpha }\right)$ is small but finite. In particular, this allows us to introduce the concept of local ergodicity [20,25], which in many realistic physical or chemical systems is much more appropriate than the global ergodicity we have considered up to now.

Here, we note that many macroscopic properties are actually only defined on the mesoscopic or continuum level. Typical examples are densities, density related quantities, and transport coefficients such as fluid densities, electric and magnetic polarizations, elastic constants, viscosity, electric conductivity, thermal conductivity, and so on [6,26]. As a consequence, values for such properties are only valid on time and length scales larger than those that define the material point in the continuum approximation. The continuum approximation implicitly assumes that we can treat the atoms contributing to the material particle as if they were locally equilibrated within the region associated with the particle; otherwise, the material point or material particle cannot be properly defined. As a consequence, the material particle exists on a finite length and time scale; in order to achieve smoothness of the system on the macroscopic level, continuum physics-based quantities are only properly defined on length scales larger than at least ten nanometers to ten micrometers (depending on the material, its phase, and the properties of interest). In order for the system to be treated as a continuum, similar estimates need to be obtained for the smallest time scales, depending on the equilibration times for the material point and the speeds with which disturbances propagate in the system; for more details, we refer to the literature [6,26]. Thus, we can usually ignore such physical properties as far as the definition of ergodicity and the conceptual developments below are concerned. However, because these properties include transport coefficients such as thermal and electrical conductivity, they play a role if one considers finite-size effects in thermodynamic models.

### 2.2. Concepts of Finite-Time Thermodynamics

The field of finite-time thermodynamics deals with the analysis of thermodynamic processes and their applications in finite time, extending to generalizations to other cost function-based processes that can be formulated somewhat analogously to thermodynamic systems [10,27]. We typically prescribe a certain total time $t_{total}$ which is available for a given process, e.g., a thermodynamic cycle (typical for extraction of work via a thermodynamic engine), or a line-type process in which the final location in thermodynamic space differs from the starting point (typical for production of a compound via a chemical reaction). When executing such a process for infinitely long process times, a total well-defined amount of, e.g., entropy is produced together with a certain amount of net work being performed or a certain amount of product produced during the chemical reactions that take place [2,3]. However, if the process is to run in finite time, usually some additional excess entropy is generated, while the amount of work extracted or the desired product generated is smaller than for the infinite-time case [28]. In the latter case of infinite time being available, one can proceed in a quasi-static fashion that is infinitely close to equilibrium, taking as much time as needed to stay close to thermodynamic equilibrium along the trajectory in thermodynamic space.

This contrasts with the FTT case, where the system never reaches true thermodynamic equilibrium for real systems along the path in thermodynamic space. However, it is usually assumed that we can stay close enough to equilibrium such that at each step it is possible to imagine (or justify) that the system relaxes close to equilibrium on some (local) relaxation time scale $\tau_{relax}$, meaning that the next step can be assumed to again start from an equilibrium state [29,30]. This already points to the issue of characteristic time scales of the real system that are relevant for a description, or at least a suitable model of the actual processes taking place during such a cycle. We note that there are exceptions to the rule that the process would ideally run infinitely slowly for an infinitely long time; for example, we might want to capture the intermediary reaction product *B* in the reaction sequence A→B→C, where an infinitely long reaction would result in total conversion of *A* to *C*.

Given some phenomenological laws or assumptions about how the finite-time condition affects the amount of (excess) entropy produced at each step, we can now perform a global optimization over the possible ways of assigning the available time to the legs of the cycle and how to allocate the time $t_{total}$ to the sub-steps within each leg. As the target of this optimization, one frequently takes the maximum work extracted within the given time $t_{total}$—or the maximum power where one employs heat and work flow (rates) instead of heat and work [31]—; alternatively, one can aim for maximum efficiency in the spirit of the original Carnot engine [14]. Furthermore, the optimization can also involve the choice of the path itself in thermodynamic space. In this case, one would perform the time allocation optimization along the path as an implicit sub-optimization step for each choice of path, although a combined one-step path-plus-allocation optimization might also be feasible in principle.

We note that these optimization or optimal control problems for a thermodynamic cycle process usually assume that the system must follow the work cycle and that it will be possible to return to the original state of the system. However, this condition is not necessarily fulfilled if we are dealing with realistic systems involving complex energy landscapes that possess metastable macroscopic states.

### 2.3. Abstract vs. Real Thermodynamic Systems

In many work cycles discussed in classical thermodynamics, it is implicitly assumed that the stability of the material or device that one employs is perfect, and that one can spend as much time as is needed (up to infinity) on each leg of the cycle, ensuring that no excess work, entropy, etc., is generated beyond the amount implied by the laws of equilibrium thermodynamics. In particular, it is assumed that global ergodicity holds, and that metastable phases or marginally ergodic material states such as glassy states or aging phenomena can be ignored. As pointed out in the previous subsection, this assumption is not always true if we are dealing with processes that run in finite time, where the system can be in non-equilibrium states for long times throughout the process. In particular, if the material employed in building the device or used as the working fluid exhibits more than one stable or metastable phase, then bifurcations during phase transitions and nucleation-and-growth processes can occur. Note that for simplicity we will use the term `working fluid’ throughout, even though the practical realization of the working fluids we discuss will often be a solid material and not a liquid or gas.

A second challenge posed by the real materials employed in devices, working fluids, or targets for thermodynamic engines and processes is the spatial dimension. It is typically assumed that we are dealing with a homogeneous material and that the changes in the environment, e.g., temperature, pressure, or volume changes, are instantaneously felt throughout the material even though they are only applied at the surface of the material. This treats the material (which in reality is macroscopic) like a small “point”, meaning that heat transport due to a change in the external temperature, propagation of pressure disturbances, etc. can be neglected as far as the analysis of the processes is concerned. Again, while such neglect can be justified for the case of the infinitely slow processes of classical thermodynamics, it cannot be justified for processes in finite time. Thus, we encounter length scales of relevance in thermodynamic engines, where the speed with which an external disturbance spreads throughout the material converts the relevant length scale into the corresponding time scale.

While heat leaks and similar phenomena have been included in FTT models of processes and cycles [32], delay times due to the spatial extent of a working fluid or target material are usually not accounted for. We note that to a certain extent such phenomena are included in the definition of the overall heat or work flow in the model systems employed in FTT as a source of irreversibility and lost efficiency, where we postulate, e.g., a law for heat conduction from the reservoir to the working fluid. In these typical endoreversible models [12] of finite-time thermodynamic processes, we might (for instance) restrict the speed with which heat is transferred to the working fluid; however, we usually do not consider the processes that are taking place inside the working fluid or their limitations and irreversibilities due to its finite size and possible internal structure. As a consequence, the assumption of endoreversibility may be too optimistic, and it is necessary to consider the details of the working fluid’s behavior during the process. Sometimes, this can be achieved by splitting the working fluid into subsystems which each can be considered endoreversible or even reversible [33,34], but this adds a lot of complexity and may not always be possible.

Clearly, the challenge is even greater if the microstructure of the material on the mesoscopic scale—single-crystal vs. granular composites vs. powder materials—becomes an issue in the performance of the thermodynamic process. One way to address this issue might be via the introduction of some “effective” or “average” properties of the material, although this might not always be feasible. Here, we need to distinguish between the use of the material as part of the device, as the working fluid, or as the target.

In the first instance, e.g., the development of a rust layer on an engine due to its exposure to the working fluid or environment, would result in limitations on the speed with which we can drive the engine when performing work without risking catastrophic failure. As a consequence, we are dealing with limitations on the time we can spend in various stages of the thermodynamic cycle, i.e., such deviations from the ideal material impose “minimum/maximum time requirements” regarding the time spent in a part of the cycle. If the material is employed as a working fluid, then the cycling through different parts of thermodynamic space will be affected by changes in the microstructure of the material as far as the speed with which the working fluid can react to, e.g., exposure to different temperatures is concerned. In this case, the various phenomenological coefficients characterizing, e.g., the heat conduction between the reservoir and the working fluid in common generic model descriptions would change from cycle to cycle, or even within a cycle. On the other hand, if the material itself is the target of the process of a chemical reaction or transformation—regardless of whether it is actually part of the working fluid or is only acted upon by the working fluid during the process—then the microstructure can be a crucial feature of the target material besides its more general atom level properties such as the composition and selected crystalline modification.

### 2.4. Concepts of Energy Landscapes

Understanding the behavior and dynamics of materials, including their stability, response to external forces, and various properties on the macroscopic, mesoscopic, and down to the atomic level, leads to the study of their potential energy landscapes as well as various generalizations such as their enthalpy or free energy landscapes. [7,8] The energy landscape of a physical or chemical system is a complex hypersurface or metagraph over a high-dimensional continuous or discrete state space, respectively. This landscape consists of microstates which underlie the microscopic dynamics of the system [7]. Each microstate *i* is assigned an energy E(i), and the space of microstates exhibits a certain topology, i.e., a neighborhood structure. This topology can represent the natural neighborhood reflecting the microscopic dynamics of the system based on the laws of physics on the microstate level. For example, for a chemical multi-atom system, a typical microstate corresponds to one specific arrangement $\vec{X}=\left({\vec{x}}_{1},...{\vec{x}}_{N}\right)$ of *N* atoms in 3D space, where ${\vec{x}}_{i}\in R^{3}$ are the coordinates of atom *i* and a neighbor microstate differs only minimally in the atom positions. Thus, the state space for a system with *N* atoms would be $R^{3N}$, and the movement of the system on the landscape can be described according to Newton’s laws via the gradient of the potential energy landscape. The topology can also explicitly define the dynamics of the system, e.g., by an exploration algorithm in a computer simulation of via probabilistic transitions among microstates reflecting the quantum mechanical nature of the system (at least partially); in such a case, we would speak of the moveclass of the system when talking about its neighborhood structure.

Regardless of whether we are dealing with a discrete or continuous energy landscape, we can now use the laws for classical or quantum mechanics to compute the time evolution of the system. However, for many systems we will notice that the time averages of observables along a trajectory of the system vary as function of the (randomly picked) starting point of the simulations, even when employing long simulation times, and can exhibit sudden jumps even after the average has seemed to converge to a fixed value.

The reason for this is that many such systems possess a multitude of metastable macroscopic states $R_{i}$, which (at least at low temperatures) can persist for very long times that often far exceed the equilibration time $\tau_{eq}\left(R_{i};a_{eq}\right)$ among all microstates that contribute to this metastable macroscopic state. As a consequence, the system appears to be in thermodynamic equilibrium for observation times $t_{obs}$ between the equilibration time $\tau_{eq}\left(R_{i};a_{eq}\right)$ and the escape time from the metastable state $\tau_{esc}\left(R_{i};b_{esc}\right)$, beyond which the likelihood of leaving the region of microstates that belong to the macrostate $R_{i}$ exceeds $b_{esc}$: $\tau_{esc}\left(R_{i};b_{esc}\right)\gg t_{obs}\gg \tau_{eq}\left(R_{i};a_{eq}\right)$ [20,25]. For these observation times, the system in the macrostate $R_{i}$ appears to be in perfect equilibrium and the laws of equilibrium thermodynamics apply to the system. For a given set of thermodynamic parameters and important physical observables, we can now determine (usually via numerical simulations of the dynamics and landscape explorations) which subregions $R_{i}$ of the (state space of the) landscape can equilibrate on a certain observational time scale while not leaving the region, i.e., we can identify the so-called locally ergodic regions which fulfill the criteria of local ergodicity by computing their equilibration and escape times [20,25].

**Figure 1:**
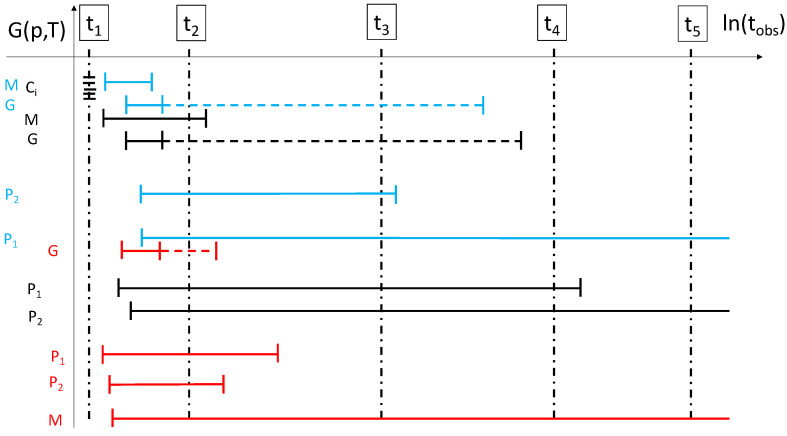
Qualitative sketch of the Gibbs free energies of some typical locally ergodic regions found in a solid for three given temperatures $T_{1}<T_{2}<T_{3}$ and standard pressure *p*, with data for $T_{1}$, $T_{2}$, and $T_{3}$ marked in blue, black, and red, respectively. Here, *C* corresponds to individual defect configurations, *M* represents the super-cooled melt for $T_{1}$ and $T_{2}$ and the actual melt for $T_{3}$, respectively, *G* is a glassy state, and $P_{1}$ and $P_{2}$ are (real, i.e., including all the local minima that correspond to equilibrium defect configurations) crystalline modifications. At temperature $T_{1}$, $P_{1}$ is the thermodynamically stable phase and $P_{2}$ is a metastable phase, just as the super-cooled melt is. The glassy state is locally ergodic only on very short time scales; for longer observation times it is only marginally ergodic and ages slowly (indicated by the dashed horizontal line). At temperature $T_{2}$, the (high-temperature) phase $P_{2}$ is now the globally ergodic equilibrium phase, while $P_{1}$ is only metastable. For both temperatures, the metastable phase and the glassy state are stable up to quite large observation times. Finally, the high temperature $T_{3}$ is above the melting temperature, and the melt is in the thermodynamic equilibrium phase. Note that the escape times from the glassy state and the crystalline phases are now quite short, even though their Gibbs free energies are much lower than at low temperatures. The vertical lines denote various observation times; e.g., at time $t_{4}$ we have only two locally ergodic regions left for temperature $T_{2}$. Remember that after preparing the system in one of the phases that are metastable for a given observation time, we will not know of the existence of competing phases with possibly lower local free energies.

In the case of chemical systems, each such locally ergodic region $R_{i}$ corresponds to a thermodynamically (meta)stable compound on a given observational time scale of interest. Thus, on short time scales the phase diagram of the system needs to include metastable phases as well [35]; only in the infinite observation time limit do we reach the classical phase diagram [7]. In particular, one can perform classical thermodynamic processes with the system restricted to this metastable compound (adding and extracting heat, applying pressures, etc.) without ever noticing that the system is not in the global thermodynamic equilibrium state, so long as the total time available for the process $t_{total}$ is much smaller than the escape time from this region $t_{total}\ll \tau_{esc}\left(R_{i};T,p\right)$ for all the values of pressure and temperature along the process path. Figure 1 shows a plot of the Gibbs free energy vs. the observation time, depicting the time ranges over which two crystalline phases, the melt, and a glassy state, are locally ergodic.

However, it should be noted that the escape and equilibration times for a metastable state will (possibly strongly) depend on the external influences that the system is exposed to. In particular, high temperatures can affect the stability of a metastable state, i.e., $\tau_{esc}\left(R_{i}\right)$ can rapidly become small when increasing the temperature. Similarly, the equilibration time will vary with temperature, but usually not as strongly as the escape time. As a consequence, a given work cycle that includes temperatures that greatly reduce the escape time must be executed quite quickly if one wants to return to the original state of the system in thermodynamic space while avoiding a phase transition into a different (meta)stable phase along the way. After all, a metastable state is by definition not in global thermodynamic equilibrium; thus, the system will not return to this state after it has left the metastable state.

In this context, we remark on the concept of marginal ergodicity [7,36,37,38]. This applies to, e.g., glassy systems and systems that exhibit aging [39,40,41,42,43]. In practice, we find for such systems that they appear to be in equilibrium for some time interval $t_{quasi}\left(i\right)$ correlated to the time since their creation $t_{age}\left(i\right)$, e.g., by quenching from a melt; this falsely suggests that the landscape region $R_{i}$ that they explore is locally ergodic. For larger times, these systems begin to show deviations from equilibrium. However, in contrast to a transition to another metastable state $R_{j}$, where the system explores and equilibrates inside a competing region $R_{j}$, here the system just explores a larger landscape region $R_{i+1}$ that encompasses the previous one ($R_{i}\subset R_{i+1}$). If we pretend that we would have started the experiment anew at the point of time after losing the equilibrium in region $R_{i}$, except now only starting our check of the equilibration after a time $t_{age}\left(i+1\right)\approx t_{age}\left(i\right)+t_{quasi}\left(i\right)$ had passed, the system would seem to be again in quasi-equilibrium, now in the larger region $R_{i+1}$. Furthermore, it would remain so for a longer time interval $t_{quasi}\left(i+1\right)$, since the time from creation $t_{age}\left(i+1\right)$ is longer than in the earlier part of the experiment ($t_{age}\left(i+1\right)>t_{age}\left(i\right)$). However, instead of stabilizing and truly equilibrating in the region $R_{i+1}$, the system again falls out of equilibrium when $t_{obs}$ exceeds $t_{age}\left(i+1\right)$, and the process repeats itself on a longer time scale. From the point of view of ergodicity, we are dealing with a sequence of ever-larger regions $R_{i}$ where $R_{i}\subset R_{i+1}$ and where the equilibration and escape time for region $R_{i}$ are approximately equal. This does not allow the system to truly enter a metastable state, while at the same time the escape time from region $R_{i}$ is approximately equal to the equilibration time of the subsequent region $R_{i+1}$: $\ldots \approx \tau_{esc}\left(R_{i+1}\right)\geq \tau_{eq}\left(R_{i+1}\right)\approx \tau_{esc}\left(R_{i}\right)\geq \tau_{eq}\left(R_{i}\right)\approx \ldots$. This process is frequently called aging, since the time in quasi-equilibrium is often more or less proportional to the time one has waited, giving the system time to “age” since its creation ($t_{quasi}\left(i\right)∼t_{age}\left(i\right)$).

Next, we briefly mention the concept of self-averaging of macroscopic systems [7,44]. It is clearly impossible to completely sample the landscape of an infinite macroscopic system on the microstate level in a finite time such that we can verify its global ergodicity with $a_{eq}=0$, and even for small finite values of $a_{eq}$ this is essentially impossible for real systems. Thus, we must ask why we can still talk about ergodicity and thermodynamic equilibrium for, e.g., a macroscopic solid, and why such systems can be employed in thermodynamic processes. Recall that classical thermodynamics usually assumes the so-called thermodynamic limit [9], where all extensive quantities of interest go to infinity while their ratios approach finite values. To address this challenge, a chimera-like approach is employed in which one represents the macroscopic system as an ensemble of essentially identical small mesoscopic systems, each of which can reach equilibrium within a finite observation time. At the same time, this set of mesoscopic systems constitutes an ensemble representing the original macroscopic system in an approximate fashion, such that the average over this ensemble’s time-averages on the time scale of observation yields a good approximation of the true ensemble average of the (infinite) macroscopic system. Clearly, such a self-average over the macroscopic solid only makes sense if the thermodynamic ground state is homogeneous (exhibiting the same atom distribution and crystalline or amorphous “structure” throughout the material); if the ground state consists of two disjoint phases with different compositions, then self-averaging is not possible (or at least only possible in the metastable phase sense discussed above).

Here, we note that by representing the time evolution of a macroscopic material in the self-averaging approximation, we are effectively constructing an approximate probabilistic evolution of the system. This type of evolution is commonly employed in (abstract) stochastic models such as Markov processes when describing the time evolution of a single (!) system in a probabilistic fashion, although the single system—defined on the microstate level of the atomistic system—should follow its own trajectory in state space that would be different from the stochastic trajectory of the probability distribution over all possible (micro)states of the system. This issue is similar to the question of time scales beyond which the results of molecular dynamics simulations agree with those of Monte Carlo random walk-based simulations [45].

Finally, we must comment on the effect of placing a system described via such a complex energy landscape in contact with an environment [7,46]. Here, we only consider the assignment of thermodynamic intensities, in particular *T* and *p*; for more examples of interactions with the environment and their inclusion in the energy landscape description, we refer to the literature [46]. Prescribing a certain temperature does not change the potential energy landscape of the otherwise isolated system, but does influence the existence, time scales, and size of the locally ergodic regions on the landscape [7]. However, the pressure and other intensities such as electromagnetic fields do affect the landscape itself by adding terms such as +pV to the energy function [7,46,47]. In particular, for a non-zero pressure we are now dealing with a potential enthalpy landscape $H_{pot}\left(\vec{X};p\right)\left(“\;=\;”E\left(\vec{X};p\right)\right)=E_{pot}\left(\vec{X}\right)+pV\left(\vec{X}\right)$ instead of a potential energy landscape $E_{pot}\left(\vec{X}\right)$. We can also visualize this as a family of such (potential energy/enthalpy) landscapes parameterized by the pressure *p*, where we switch to neighbor landscapes when changing the pressure. Each of these enthalpy landscapes exhibits its own locally ergodic regions, which correspond to, e.g., high-pressure phases or phases that are (meta)stable at effective negative pressures. In particular, a thermodynamically stable modification can become metastable or unstable in response to a change in pressure; thus, in searching for metastable modifications of a material, one explores not only the potential energy landscape but also a wide spectrum of potential enthalpy landscapes for both negative and positive pressures [48].

In the chemical and physical literature, the complex energy landscapes of material systems have been frequently employed to study (thermodynamic) behavior, particularly of liquids [24,49,50,51,52] and glasses [53,54,55,56,57,58] (e.g., polymers [39,59,60,61,62,63,64,65], solids [66,67], spin systems [68,69,70,71], etc.) as well as proteins and biopolymers [61,72,73,74,75,76,77,78,79], where the complexity of the barrier structure of the multi-minima landscape is expected to prevent the system from reaching global thermodynamic equilibrium. In the case of glassy materials, this inability to equilibrate and the multitude of competing more-or-less disordered local minima on the energy landscape constitutes nearly the defining quality of a glass. A major goal of such studies has been to understand the way in which such materials can be fitted into the classical phase diagram picture of matter, together with explaining their unusual long-time dynamics exhibiting phenomena such as aging [39,40,80,81] which do not appear in the usual ordered crystalline phases.

Furthermore, the ability of glasses to very rapidly become less viscous over a rather small temperature range that remains below the melting point of the (crystalline) material (the converse of the nearly exponential rise in viscosity upon quenching the glass-forming melt [82]) remains a puzzling aspect of glassy systems. A possible answer has been proposed based on the observation that many glass-forming systems exhibit an exponentially growing local density of states [55,71,83,84]. It can be noted that for such systems we observe a bifurcation behavior called “exponential trapping” when one computes the probability of the system residing in a given range of energies as a function of the temperature [84,85,86]. The system is either deep inside the local minima basins, meaning that it faces large barriers against transformation into energetically more favorable states (the highly viscous quasi-equilibrium glassy state), or is found at the top of the energy range for which the local densities of states are growing exponentially. In this case, the system only needs to cross much lower energy barriers to neighbor basins with a concurrent much higher mobility of the atoms, i.e., we are now dealing with a low-viscosity melted material. Here, the fascinating aspect is that the range of temperatures over which the switch occurs between these two different (marginally and locally ergodic, respectively) regions of the energy landscape with very distinct rheological behavior is very small regardless of the difference in energy between the top and the bottom of the part of the landscape exhibiting the exponentially growing densities of state.

This is in contrast to the case of proteins, where a main challenge has been understanding the ability of certain proteins to reach their native folded state on comparatively short time scales in spite of their complex landscape, in something like the converse of the glass transition problem. One popular answer to this so-called Levinthal’s paradox [72] is the assumption that the energy landscape exhibits a funnel-like structure for these specific proteins [75,87], with rather low energy barriers to neighboring local minima but a clear trend for the pathways on the landscape to guide the probability flow towards the low-energy region. This allows the protein system to reach this region of the landscape corresponding to the native state in spite of the multitude of competing minima which might otherwise trap the system along paths where the large majority of accessible paths lead to the native state.

These issues and other related ones have been extensively discussed in the literature over the past sixty years. For general multi-minima systems in chemistry such as crystals [48,88,89,90,91,92,93,94,95,96,97,98,99,100,101], clusters [102,103,104,105,106,107,108,109,110,111,112], and complex molecules [104,113,114,115,116,117,118], their complex landscapes have been explored mainly with the goal of identifying promising (meta)stable modifications and isomers. Furthermore, the free energies have been computed for individual locally ergodic regions, usually in the harmonic approximation [119], and (tree) models for so-called free energy landscapes have been constructed [120,121,122] (“so-called” because the issues of the relevant timescales for the existence of the locally ergodic regions and the free energy barriers between them are typically ignored in their construction [123]). Finally, equilibration trees have been computed that indicate the way in which different sub-regions of the energy landscape equilibrate into larger locally ergodic regions on the level of both microstates [71,83] and minima [124].

These are fascinating research questions in themselves, and have formed the motivation behind the development of many algorithms for studying complex energy landscapes. They have also inspired many concepts and model descriptions of such landscapes; for an overview, see [7,8]. However, although issues such as the glass transition and the folding pathways on biologically relevant timescales highlight the finite-time aspect as part of their formulation, we are not going to discuss these phenomena in this perspective, as the focus here is on classical thermodynamic processes and the optimization of their output in finite time, be it work or heat production and transfer without wasting work or generating excess entropy, the efficiency of such processes, or the amount of material that can be produced with minimal effort.

## 3. Prototypical Examples

This section briefly discusses prototypical examples of classes of processes for systems where the complex energy landscape adds features that need to be taken into account when setting up (and subsequently solving) the optimization problems of finite-time thermodynamics. For every class of systems, we will point out the time scales of relevance that need to be considered, the quantities one might want to optimize, the type of cycles that are of interest, and the special complications that can arise when modeling and optimizing the relevant processes. Given this focus on the additional features and challenges due to the complex energy landscape involved, we will not discuss standard aspects of the FTT description of thermodynamic processes and their optimization, referring to the literature for such details [10,11,13,27,125]. Similarly, we do not include finite-time studies of thermodynamic engines where a phase transition occurs as part of the cycle but where the actual transformation process is of no major concern, and is dealt with in a generic way as part of the overall heat transfer along the leg where the (isothermal) transition occurs [126].

These descriptions are only rough schematics, and are only meant to provide guidance for dealing with actual problems of interest; realizing and solving such an example in practice is a whole research project in itself [127,128], and goes beyond the purview of this concept-focused presentation.

### 3.1. Thermodynamic Processes Involving Phase Transitions

The most relevant class of examples for FTT processes involving systems with complex energy landscapes concerns the use of materials as working fluids which can exhibit more than one phase during operation of the engine along its path in thermodynamic space. In a more general sense, this also includes the use of such materials in building the device, as already discussed in Section 2.3, where possible complications due to wear and tear of the real device were noted together with the limitations on the speed with which the thermodynamic intensities *T* and *p* can spread throughout the working fluid. Such issues can affect the efficiency and capability of the device, e.g., as limitations on the speed at which the thermodynamic cycles can be performed.

We note that the pure “mathematical thermodynamicist” who wants to focus on analytically solvable problems might prefer to consider issues concerning metastability, instability, irreversibility, etc., as an external mathematical side-condition on the optimization problem, such as adding heat conduction between hot and cold reservoirs leading to leakage of heat from the system proportional to the time spent in the overall cycle. Still, even such a researcher needs to first formulate a mathematical model of this feature that represents real-life engines and working fluids, before such real-life aspects can afterwards be reduced to a formal overall factor encapsulating a high entropy production rate or loss of work at certain parts of the cycle.

#### 3.1.1. No Competing Crystalline/Amorphous Phases

**Figure 2:**
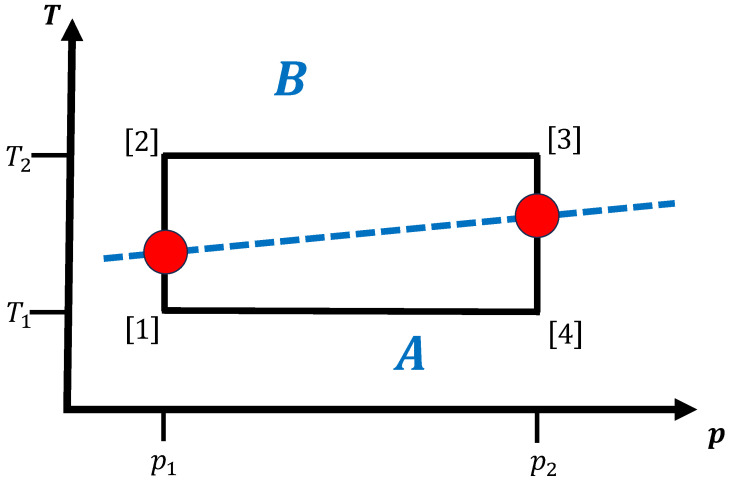
Sketch of a simple cyclic path ([1]→[2]→[3]→[4]→[1]) in (T,p) space, with two phase transitions: from equilibrium phase *A* to equilibrium phase *B* in the first leg, and from phase *B* to phase *A* in the third leg, respectively. The dashed blue line is the equilibrium phase separation line in the (T,p) phase diagram. The points where the transition would occur for an infinitely slow cycle are indicated by red spots.

The simplest case is a system with a working fluid that consists of a homogeneous (infinitely large) material that can exist in several equilibrium phases but where neither metastable modifications nor amorphous/glassy states are present. For each point in thermodynamic space (T,p,…), only one phase is present, i.e., on all observational time scales of interest, the energy/enthalpy landscape exhibits only one locally ergodic region, which is identical with the globally ergodic equilibrium phase for this temperature and pressure. For simplicity, we assume that only two equilibrium phases *A* and *B* exist in the region of thermodynamic space where the cyclic or line-type path resides; here, we denote a path to be a line-type one if the starting and end points are different and if the path does not intersect itself. Figure 2 shows a simple cyclic path in (T,p) space, where for low and high temperatures the thermodynamic equilibrium phases are *A* and *B*, respectively.

The goal of the thermodynamic process is to perform some work in a finite time $t_{total}$, where the excess entropy/heat production or loss of work due to the finite-time requirement is minimized or the efficiency of the process is maximized. For simplicity, we assume a cyclic process with a prescribed path in (T,p) space, where the time spent in each leg of the path and the way in which the time is allocated along the legs are subject to the optimization. Along this cyclic path, the material switches between two (or more) equilibrium phases, such that at least two phase transitions will occur. We note that of course the case of a path with only one transition that can occur when cycling around a second order phase transition point in (T,p) space—e.g., in some cycles involving a gas–liquid transition—also works, and might be of interest in certain kinds of refrigeration cycles employing the gas–liquid phase transition. In this context, we recall that the phase transitions occur along lines or at points in thermodynamic (T,p) space; depending on which value is varied, one can speak of transitions driven mainly by pressure changes or mainly by temperature changes. In the kinds of materials we are discussing here, most of the transitions are first-order, although second-order transitions can also occur. In solids, the latter are mostly associated with small displacements of the atoms changing the symmetry group of the crystal to a subgroup, such that no nucleation of the new phase followed by growth of the new phase from this nucleus is needed. As a consequence, for second-order phase transitions there is no free energy barrier for the atom rearrangement, and the amount of thermodynamic work needed is small. Of course, if the transformation takes place at points in (T,p) space that are some distance away from the second order transition point or line, then we are dealing with a first-order transition.

**Figure 3:**
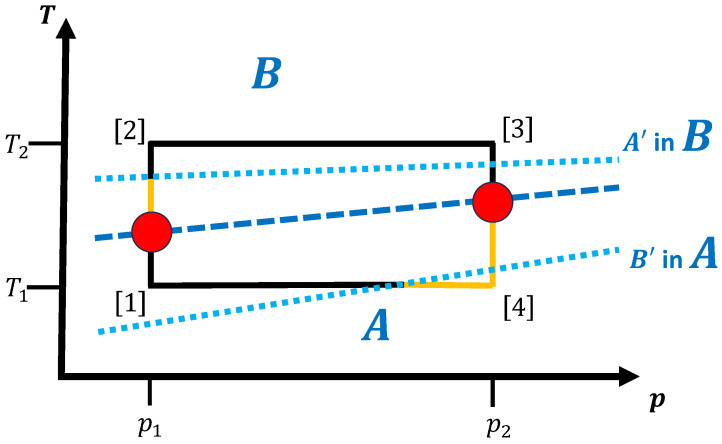
Sketch of a simple schematic path in (T,p) space with two phase transitions: from equilibrium phase *A* to equilibrium phase *B* in the first leg, and from phase *B* to phase *A* in the third leg, respectively. The dashed dark blue line is the equilibrium phase separation line in the (T,p) phase diagram, while the dotted light blue lines indicate the range in (T,p) space where the metastable phase (marked as $A^{\prime }$ and $B^{\prime }$) is still locally ergodic on moderately large time scales. The points where the transition would occur for an infinitely slow cycle are indicated by red spots, and the orange colored part of the path shows the range of the path where “non-trivial” times would be needed to perform the transition from the metastable phase to the equilibrium phase in the event that only a finite time $t_{total}$ is available for running the whole cycle. Note that the difficulty of nucleating phase *A* from the metastable phase $B^{\prime }$ extends to the fourth leg of the cycle, even though the stability of phase $B^{\prime }$ is greatly reduced around point [4], which marks the high-pressure and low-temperature region along the path. On the other hand, after the transition has taken place, the metastable phase becomes essentially inaccessible. Typically, the farther away we are from the actual equilibrium phase transition line, the faster the nucleation-and-growth transition can take place. However, we note that the actual length of the orange sections of the path would depend on the speed at which we are moving along the path, relative to which transition times could be considered “non-trivial”; the more slowly we can move along the cycle overall, i.e., the larger $t_{total}$, the shorter the orange sections will be. This can lead to confusion if we were to interpret the orange color as indicating the section of the path where we still observe the system in the respective metastable phase; such an interpretation would only be appropriate for a given specific choice of time allocation along the path.

Upon a change in temperature and/or pressure at the transition temperature/pressure along the path, the material has to re-organize on the atomic level in order to transform into the new equilibrium phase. To achieve this process in finite time, it is usually necessary to move the system to temperatures/pressures deviating from the equilibrium transition values such that the material enters a (potentially massively) non-equilibrium state. In this state, the first phase is no longer stable, but the new equilibrium phase is not yet established; in order for the system to settle into this new phase within a finite time, excess entropy/heat generation or extra work is required. Figure 3 shows another schematic cycle, where we now indicate the ranges of metastability of phases *A* and *B* in the respective regions where phases *B* and *A* are the equilibrium phases.

In order to design models of this process for the purpose of time-allocation optimization, we note the relevant time scales involved. Obviously, there is the available time for the whole cycle $t_{total}$, which distinguishes finite-time thermodynamics from classical thermodynamics. Next, we discuss various equilibration time scales; note that in the following we do not worry about $a_{eq}$ or about the observables $O_{\alpha }$ used to classify ergodicity and equilibrium. The first time scale of interest is the equilibration time $\tau_{eq}\left(i;T,p\right)$ for phase *i* when starting from a generic point inside phase *i* for general values of *T* and *p* for which phase *i* is locally ergodic. The second time scale is the relaxation time $\tau_{relax}\left(i;T_{new}-T,p_{new}-p\right)$ for phase *i* after a (small) perturbation of the system away from equilibrium. Here, we are starting from a point on the landscape belonging to the locally ergodic region $R_{i}$ of the landscape associated with the system in equilibrium at (T,p), except that now the thermodynamic parameters have been shifted to (nearby) values $\left(T_{new},p_{new}\right)$, placing this point at the rim or even slightly outside the new locally ergodic region associated with the thermodynamic point $\left(T_{new},p_{new}\right)$. Although strictly speaking being an equilibration time, $\tau_{relax}$ corresponds to the usual relaxation time to equilibrium that is employed when modeling and optimizing the time step allocation for a standard working fluid that needs to be kept in or at least very close to equilibrium. Typically, one assumes that some simplified (phenomenological) model can be used for the excess entropy production as long as the time step associated with moving from (T,p) to $\left(T_{new},p_{new}\right)$ is larger than $\tau_{eq}\left(i;T_{new},p_{new}\right)$.

The third group of time scales concerns those associated with the phase transition A→B. The first is the equilibration time inside the locally ergodic region associated with phase *B*, $\tau_{eq}\left(B;T,p\right)$. Next, there is the escape time from inside the unstable/metastable phase *A*, $\tau_{esc}\left(A;T,p\right)$. However, for complex energy landscapes it is not obvious that leaving region *A* will automatically transfer the system at once into the neighborhood of region *B*; instead, the system enters a general transition region that connects regions *A* and *B*. This region can be quite large, and in general may border on many other (locally ergodic or marginally ergodic) regions of the landscape. In particular, reaching the right “exit” from such a complex transition region crossing all of the generalized barriers involved can require quite a long time [38]. Thus, the escape time from region *A* together with the movement of the system inside the transition region into the neighborhood of the (stable) target phase *B*, $\tau_{esc}\left(A\to B;T,p\right)$ at the point (T,p) in thermodynamic space where phase *B* is the equilibrium phase can be considerably larger than $\tau_{esc}\left(A;T,p\right)$. Here, we note that during the subsequent equilibration “into” phase *B* when starting from the region of the landscape formerly associated with phase *A* or the transition region connecting *A* and *B*, the system does not start from a generic point inside phase *B*, as is usually assumed in the definition of $\tau_{eq}\left(B;T,p\right)$; instead, we start from a very specific point on the landscape inside the region associated with the former locally ergodic phase *A* or the transition region surrounding it. Thus, we denote this time scale by $\tau_{eq}\left(A\to B;T,p\right)$. Therefore, $\tau_{eq}\left(B;T,p\right)$ will usually be smaller than the time required to move from the exit of the transition region into the locally ergodic region *B* and equilibrate there, i.e., $\tau_{eq}\left(A\to B;T,p\right)>\tau_{eq}\left(B;T,p\right)$. These two time scales $\tau_{eq}\left(B;T,p\right)<\tau_{eq}\left(A\to B;T,p\right)$ and $\tau_{esc}\left(A;T,p\right)<\tau_{esc}\left(A\to B;T,p\right)$ constitute lower bounds on the time scale within which a successful transition from the newly unstable phase *A* into the new equilibrium phase B takes place on the energy landscape.

If the system in phase *A* still has a certain degree of stability, as is often the case in first order phase transitions, it can be considered locally ergodic on some small time scale; thus, the quantity of interest would usually be this escape time $\tau_{esc}\left(A\to B;T,p\right)>\tau_{eq}\left(A\to B;T,p\right)$. On the other hand, if the system can no longer equilibrate on essentially any time scale of interest in phase *A* at point (T,p) in thermodynamic space, then the equilibration time of the new phase would often be the relevant quantity, i.e., $\tau_{esc}\left(A\to B;T,p\right)<\tau_{eq}\left(A\to B;T,p\right)$.

A special very important case in this context consists of nucleation-and-growth processes, which proceed in two stages: the formation of a stable nucleus of phase *B* inside the metastable phase *A*, with its time scale $t_{n}\left(B\;\mathrm{in}\;A;T,p\right)$ (a special case of $\tau_{esc}\left(A\to B;T,p\right)$), and the growth process of this nucleus into the macroscopic phase *B* on the time scale $t_{g}\left(B\;\mathrm{in}\;A;T,p\right)$ (a special case of $\tau_{eq}\left(A\to B;T,p\right)$). We note that the system might well have left the locally ergodic region *A* before the nucleus of phase *B* has come into existence, i.e., $t_{n}\left(B\;\mathrm{in}\;A;T,p\right)\gg \tau_{esc}\left(A;T,p\right)$. Furthermore, during first-order phase transitions, $t_{g}\left(B\;\mathrm{in}\;A;T,p\right)$ can far exceed $\tau_{eq}\left(B;T,p\right)$; thus, the total time $t_{n+g}\left(A\to B;T,p\right)$ needed for such a process can greatly exceed the sum of $\tau_{esc}\left(A;T,p\right)$ and $\tau_{eq}\left(B;T,p\right)$, $t_{n+g}\left(A\to B;T,p\right)=t_{n}\left(B\;\mathrm{in}\;A;T,p\right)+t_{g}\left(B\;\mathrm{in}\;A;T,p\right)\;\left(\approx \tau_{esc}\left(A\to B;T,p\right)+\tau_{eq}\left(A\to B;T,p\right)\right)>\tau_{esc}\left(A;T,p\right)+\tau_{eq}\left(B;T,p\right)$.

Of course, we assume that a phase transition occurs in the first place: if $\tau_{esc}\left(A;b_{esc}\right)\gg \;t_{total}$ for the starting phase A for all values of (T,p) along the path, then no transition happens during the cycle and we stay in the same locally ergodic region *A* as far as the material is concerned. Analogously, if $\tau_{esc}\left(B;b_{esc}\right)\gg t_{total}$, then the transition back to the initial phase will not take place, and we are stuck in the second phase at the end of the cycle. In this case, it is necessary to add a long waiting time before restarting the cycle in order to allow the system to re-initialize and reach equilibrium in phase *A*. For a cycle that is performed periodically, this waiting time clearly poses a problem. An alternative to deal with either of these events within the available time $t_{total}$ can be the choice of a different cycle, e.g., extending to higher or lower temperatures and/or different pressures, where the escape times from phases *A* and *B* are much shorter and the cycle can be performed as desired, though at the expense of a much higher excess entropy production or loss of work.

A related problem can occur if $\tau_{eq}\left(A\to B;T,p\right)\gg t_{total}$ for the whole region of the path in (T,p) space where phase *B* is the equilibrium phase. In this instance, the working fluid remains in a non-equilibrium state for the whole duration of this stage of the path, never quite leaving the transition region connecting regions *A* and *B*, and we need to explicitly model the thermodynamic processes during this stage on a non-equilibrium basis. After entering the stage of the path where *A* is again the equilibrium phase, the problem might be alleviated, since it is to be expected that the relaxation back to the phase *A* might be rather fast and within the time limits of the process. Again, we can try to address this issue by choosing a different thermodynamic path, along which the two phase transitions occurring quickly enough.

In this context, we recall the probabilistic aspect of the escape time definition, incorporated in the characteristic constant $b_{esc}$: if we attempt the transition $1/b_{esc}$ times, each time running the simulation/experiment for a length of $\tau_{esc}\left(b_{esc}\right)$, then we expect that we will make a transition into the other phase at least once. In order to take this feature into account, a probabilistic approach can be employed to model the behavior of the system along the cycle, as outlined below in Section 3.1.5.

Assuming that neither of these serious problem cases occur, it is nevertheless clear that we need to spend enough time in the phase transition region to reach the new phase *j* from *i*, i.e., along these legs of the path, the times $t_{n+g}\left(i\to j;T,p\right)$ need to be included and subtracted from the total available time $t_{total}$, and the excess heat or entropy produced during the nucleation-and-growth stage must also be accounted for. As mentioned above, in order to speed up the phase transformation process, we might have to employ values of (T,p) that are (possibly considerably) larger/smaller than the values where the two phases are in equilibrium. Depending on how much time we can afford to spend on the phase transition, we can reduce the excess entropy production by staying closer to the transition values of pressure and temperature. On the other hand, spending too much time at the phase transition forces us to be “too fast” on the rest of the path, again producing excess entropy. One critical issue in the modeling of such rather fast movements along the cycle is that we might move the system so far out of equilibrium that the linear approximation-based models for the entropy production during relaxation to equilibrium (frequently used when computing or estimating optimal schedules [29]) are no longer applicable, considerably complicating the modeling of the thermodynamic cycle. This usual (excess) entropy production along the path in thermodynamic space and its finite-time optimization is not discussed here; we assume that we know or can model the excess entropy production due to the usual finite-time movement along the path for a given material once it is essentially equilibrated inside one phase, since this constitutes the standard FTT problem.

A detailed prototypical example of modeling a first-order phase transition using a finite-time optimal control analysis can be found in the study of the liquid–gas transition by Santoro et al. [127]. There, the authors minimized the excess work needed to perform this phase transition in a finite time slightly away from the phase transition point in thermodynamic space. Their study included explicit models for the nucleation and growth processes. Such models would also be necessary for the study of any thermodynamic cycle that includes phase transitions if one desires to obtain quantitative results for the optimization of a specific phase transition in a given chemical or physical system.

#### 3.1.2. Presence of Amorphous Precursor or Glassy States

In the preceding Section 3.1.1, we have considered the case of a system with only two phases, which transform along a certain line in thermodynamic (T,p) space into each other. For first-order phase transitions A→B, the transition is not instantaneous but commonly takes place through a nucleation-and-growth process on a time scale $t_{n+g}\left(A\to B;T,p\right)$ that depends on the thermodynamic conditions (T,p). For such processes, we deal with free energy barriers that require a certain time to cross in order to create a nucleus of critical size large enough to grow into the new phase [129,130,131]. In order to achieve this in finite time, we must move beyond the transition line in (T,p) space deeper into the region where phase *B* is the equilibrium phase, which requires excess work or excess entropy. For simplicity, we can visualize the nucleation process being characterized by the above-mentioned escape time $\tau_{esc}\left(A;T,p\right)$ from phase *A*, while the time spent in the growth stage of the transition is related to the equilibration time scale $\tau_{eq}\left(B;T,p\right)$ in phase *B*. We have noted already that $\tau_{eq}\left(B;T,p\right)$ is only a lower limit on the time that this growth process can take, as we do not start from a generic point inside the locally ergodic region *B* on the energy landscape. As a consequence, as already mentioned earlier, the overall nucleation-plus-growth time scale usually exceeds the sum of the above two time scales, $t_{n+g}\left(A\to B;T,p\right)>\tau_{esc}\left(A;T,p\right)+\tau_{eq}\left(B;T,p\right)$.

In practice the situation is more complex, since the nucleation process takes place via the generation of a multitude of nuclei throughout the macroscopic material. The precise number depends on the specific values of the temperature and pressure applied, quite frequently leading to the formation of a polycrystalline material with less well defined macroscopic properties instead of a single crystal. Furthermore, during a general nucleation process the system resides in a transition region on the complex energy landscape, from which in principle several alternative (metastable) phases can be reached, assuming that such additional phases exist on the landscape in the first place.

In particular, in many materials such a transition region—while never being locally ergodic in itself, and as such not corresponding to a thermodynamic (meta)stable phase—is structurally realized in solid form as an amorphous precursor state AP or a glassy state *G*, which can persist in some fashion for quite a long time at low enough temperatures. We note that there are of course many amorphous solids with surprisingly high stability that can be created employing a variety of alternative routes; the generation of amorphous Si_3_B_3_N_7_ via the sol–gel process [132,133] constitutes such a case. However, here we only focus on those amorphous materials that are generated along a thermodynamic path, i.e., via quenching from a melt or the gas phase, or via amorphization through high-pressure treatments.

In the general amorphous state, the material is far from global thermodynamic equilibrium and serves as the matrix within which thermodynamically (meta)stable crystalline phases *i* can nucleate. This synthesis approach of crystallization from an amorphous precursor is frequently employed to access unusual crystalline modifications from, e.g., an amorphous film deposited from the gas phase, or to reach a high-pressure phase from a material that is being amorphized as an intermediary stage via application of high pressures and temperatures. The corresponding time scale for the specific nucleation-and growth process $t_{n+g}\left(AP\to i;T,p\right)$ now takes the place of the earlier mentioned time scales—$\tau_{esc}\left(i\right)$, $\tau_{eq}\left(j\right)$, and $t_{n+g}\left(i\to j\right)$ during first-order phase transitions—and must be compared with the total time available for the cyclic or line-type thermodynamic path. We note that the ability to access multiple metastable phases from such a precursor can be an advantage if one wants to reach valuable or just interesting phases that might not be accessible otherwise [134,135,136]. However, this adds uncertainty to the process with respect to its outcome, in addition to the excess work/entropy associated with the phase transition in finite time. To deal with this uncertainty, we need to introduce a probabilistic scheme into the cycle description, which is described in more detail in Section 3.1.5.

In many regards, the glassy state *G* can be treated as another instance of the amorphous state, although it exhibits some features that are of particular relevance for the thermodynamic cycles that include phase transitions which we are discussing here. Similar to the amorphous state, the glassy material will also eventually transform into the thermodynamically more stable equilibrium phase via some kind of nucleation and growth process. However, the glassy state tends to be quite stable on long time scales as far as its macroscopic properties are concerned. This also often applies to its general structural features; in particular, both the time scale of nucleation and the time scale for growth of a crystalline phase *i* inside the glassy state *G*, $t_{n}\left(i\;\mathrm{in}\;G;T,p\right)$ and $t_{g}\left(i\;\mathrm{in}\;G;T,p\right)$, respectively, can be very large, and may often exceed the total time available for the cycle, $t_{total}<t_{n}\left(i\;\mathrm{in}\;G;T,p\right)$, $t_{g}\left(i\;\mathrm{in}\;G;T,p\right)$.

This is related to the fact that in many materials of interest, glassy states appear when quenching a melt (i.e., cooling the material too fast to a temperature below the freezing temperature for nucleation of the crystalline equilibrium phase to occur). As a consequence, the glassy material resembles a liquid with extremely high viscosity in many aspects. Here, we note that the glassy state should not be confused with a metastable super-cooled liquid; the moment the super-cooled liquid experiences a (local) disturbance above a certain (small) strength, a spontaneous nucleation of many nuclei of the solid equilibrium phase takes place and these nuclei very rapidly grow to produce a polycrystalline material. In contrast, the glassy material is very stable, and even a strong disturbance like being hit with a hammer only results in mechanical damage, not in the initiation of a nucleation and growth process. As a consequence of being a quasi-equilibrium continuation of the melt in the solid state, such glassy systems typically exhibit marginal ergodicity and show aging, as discussed in Section 2.4.

**Figure 4:**
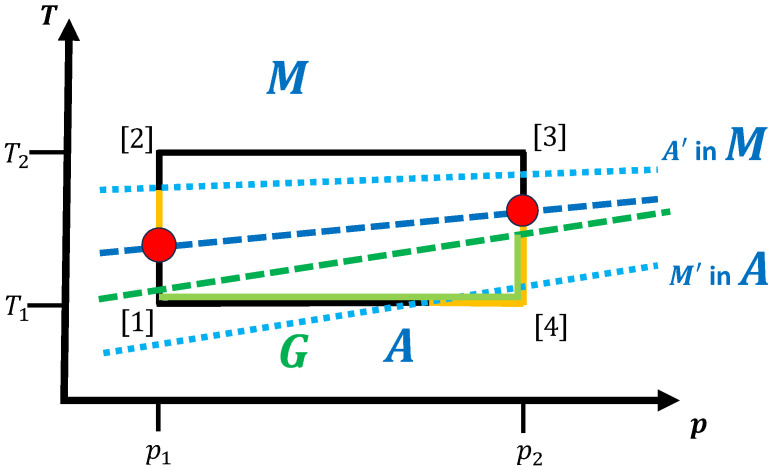
Sketch of a simple schematic path in (T,p) space with two phase transitions: from the crystalline equilibrium phase *A* to the melt equilibrium phase *M* in the first leg, and from phase *M* to phase *A* in the third leg, respectively. The dashed dark blue line is the equilibrium phase separation line in the (T,p) phase diagram between the melt and the solid, while the dotted light blue lines indicate the range in (T,p) space where the metastable phase (marked as $A^{\prime }$ and $M^{\prime }$) is still locally ergodic on moderately large time scales. The points where the transition would occur for an infinitely slow cycle are indicated by red spots, while the orange colored part of the path shows the range of the path where “non-trivial” times would be needed to perform the transition from the metastable super-cooled melt or super-heated crystalline phase to the crystalline or melt equilibrium phase, respectively, when only a finite time $t_{total}$ is available for running the whole cycle. As an added complication, the system is assumed to be a glass-forming one, meaning that the glassy state can exist on long time scales (below the dashed dark green line) in competition with the crystalline equilibrium phase. If the nucleation and growth process of phase *A* from the melt does not take place along the thermodynamic path, e.g., because we are moving too quickly through the phase transition region close to the phase transition point on leg 3 of the cycle, then the system will exhibit a glass transition and remain in the glassy state for the rest of the cycle. This is indicated by the light green path in (T,p) space, which begins slightly below the equilibrium phase transition point on the third leg of the cycle when the glassy state becomes long-time stable, and persists until the ending/starting point [1] of the cycle has been reached.

For the thermodynamic cycles in finite time that we are interested in, such glassy states can, e.g., occur if the two phases that are visited along the trajectory in (T,p) space correspond to the solid crystalline modification (*A*) and the melt (*B*). Melting a solid crystalline phase is usually quite straightforward as long as only one solid phase exists in the system close to the melting temperature, and this melting transition usually takes place relatively quickly even for temperatures close to the melting point. We note that some complications can occur for unusual systems such as gallium [137]; however, here we focus on the standard case. Furthermore, the formation of the thermodynamically stable crystalline solid phase from the melt when cooling the system usually requires a non-negligible amount of time even if the material is not a glass former. As mentioned above, if glassy states *G* in the system are possible or likely, then the time to reach the crystalline modification *A*, $t_{n+g}\left(G\to A;T,p\right)=t_{n}\left(A\;\mathrm{in}\;G;T,p\right)+t_{g}\left(A\;\mathrm{in}\;G;T,p\right)$ can be very large, far exceeding the equilibration time of the crystalline modification $t_{n+g}\left(G\to A;T,p\right)\gg \tau_{eq}\left(A;T,p\right)$, and might require careful tuning of the thermodynamic conditions. Figure 4 shows the simple cycle already discussed in Figure 2 and Figure 3, except that now phase *B* is the melt (denoted by *M*) and we encounter the added complication that the system can enter a glassy state from the melt instead of nucleating into the equilibrium phase *A*.

For the purposes of our discussion, this implies that if $t_{n+g}\left(G\to A;T,p\right)$ exceeds $t_{total}$, then we will remain in the glassy state of the material for the rest of the trajectory. In particular, if the cycle is run with a starting point in the crystalline phase *A*, then we do not reach the original starting point of the cycle, and not only in a small fraction of instances; i.e., being stranded in the glassy state is not a low-probability occurrence. If the working fluid at the start of the cycle had been the melt *M*, i.e., if the cycle were to have started at point [2], and if the cycle included a transformation into the solid phase *A* before returning back to the melt, then the situation is problematic but not completely lost. In this latter case, the solid phase that we access and perform some of the work and heat transfer with is just the glassy state of the material instead of the crystalline equilibrium phase. As long as the properties of the glassy material are such that all relevant tasks can be performed—although perhaps not with the same efficiency compared to using the crystalline state as the working fluid—then the thermodynamic cycle can still be completed.

In principle, we could choose the glassy state and the melt as the two “phases” of interest, since then we can complete the cycle, seemingly returning the working fluid to its original state at point [1]. Nevertheless, even in this case we have to deal with the aging of the glass that will occur while the material of the working fluid is in the glassy state. Depending on the length of time for which the material remains a glass, it will slowly evolve; along each point of the trajectory in (T,p) space, the system “quickly” leaves the marginal quasi-equilibrium state reached for these thermodynamic conditions and continues to respectively “emit” or “collect” (configurational) entropy into or from the universe as time goes on, while approaching (though not reaching) the crystalline equilibrium phase. Here, we note that this change in configurational entropy occurs in addition to the usual excess entropy associated with the usual “equilibration” that we observe in the equilibrium solid (crystalline) phase when perturbed (slightly) out of equilibrium. As a consequence, if we want to use the glassy state as the starting state of the cycle together with the melt as the second phase—a cycle which can be achieved, since melting a glass is no more problematic than melting a crystal—then we have to realize that the final glassy state is unlikely to be in exactly the same thermodynamic state as the starting one, which presumably had been relaxed for a long time before the engine was started. Nevertheless, if we immediately restart the engine after finishing one cycle, then after a few cycles the material will be in the “same” (evolving) glassy state of the same age at point [1] from one cycle to the next.

Here, we note that such a very slow approach to the equilibrium solid state can also appear in systems where the high-temperature phase corresponds to a solid solution instead of the melt and where the solid equilibrium phase at lower temperatures corresponds to a separation into two solids with different concentrations (possibly realized in a poly-crystalline fashion). In particular, if the solid solution state is quenched to a temperature much below the critical point of the miscibility gap in the phase diagram of the material, where the thermodynamically stable phase would consist of two separated solid solutions with different compositions [2,138], then it can occur that the system very slowly evolves into the final two-solid phase. As a consequence, the material exhibits a state between a simple homogeneous metastable solid solution and the equilibrium state consisting of two segregated solids with different compositions, each of which is in thermodynamic equilibrium by itself. Conversely, the same can happen if we raise the temperature on the two-solid phase and have to wait a long time before the thermodynamically stable homogeneous solid solution equilibrium phase has been able to form at the higher temperature. Here, one should keep in mind that the solid solution phase is also a solid; thus, the internal atom diffusion needed to establish the equilibrated solid solution phase is likely to proceed quite slowly. Hence, we observe that returning to the original state of the material can become extremely difficult. We need to spend a great deal of time at the right transformation conditions (temperature, pressure, etc.) in order to nucleate the thermodynamically stable phase out of the glassy, amorphous, or solid solution state. This might force us to add a whole thermodynamic cycle or cycles after our original work cycle in order to re-establish the original starting phase of the working fluid material.

In this context, we note that even if we do not transform the crystalline starting material into the liquid state (from where the subsequent transformation into the glassy state would occur) because we stay below the melting temperature along the whole path, softening (or hardening) can still take place when approaching the melting temperatures/transformation pressures, with cyclic fatigue phenomena appearing [139]. In this case, high amounts of disorder can arise in the single-crystal material, which can result in both microscopic and mesoscopic structural changes. Similarly, high pressures, even when quite far below the actual transition pressures needed for the formation of a high-pressure phase, can change the amount and distribution of equilibrium and non-equilibrium defects, and create domain changes which take considerable effort and time to reverse. This is particularly critical if the properties of the material (electronic, mechanical, etc.) actually depend on the number and distribution of the (equilibrium and non-equilibrium) “defects” in the solid.

Quite generally, in many materials we find slow weakening or other changes that can occur during thermodynamic cycles and need to be accounted for during computation of the efficiency of the cycle and the resulting excess entropy or loss of available work. These are especially important if they concern the mesoscopic structure (crystallite size, domain size, grain boundary and dislocation distribution, interfaces of composite materials), as these tend to be more difficult to reverse. This applies even if no competing metastable phases are present. In particular, we acquire large numbers of defects or other changes which are not eliminated upon return to the starting material, even if it is still a single-crystal or polycrystalline material of the only solid phase that is locally ergodic in the system. One cannot magically make it so without spending enormous amounts of effort and work on essentially “re-forming”/“transforming” the material at the end of the cycle back into the original starting equilibrium material. We might treat these large re-initializations of the working fluid as somewhat external features one would prefer to ignore when designing and optimizing a work cycle. Nevertheless, such additional work and entropy production needs to be accounted for when analyzing the finite-time thermodynamics of systems that employ materials exhibiting long-lived amorphous or glassy states as working fluids.

#### 3.1.3. Existence of Multiple Metastable Phases in Parallel

Clearly, the situation is even more critical if the material possesses several competing moderately long-lived metastable phases *i* at (T,p) values along the path of the thermodynamic cycle. In contrast to the case of the glassy state, which is expected to vary slowly while inexorably transforming more and more into the equilibrium phase, we now deal with well-defined phases that are in local equilibrium on time scales of interest along the cycle, i.e., once we are in such a phase *i*, equilibrium thermodynamics holds on time scales $\tau_{eq}\left(i;T,p\right)\ll t_{obs}\ll \tau_{esc}\left(i;T,p\right)$. In the following, we distinguish two main cases: where we start the cycle with the working fluid in the equilibrium phase (case 1), and where we start with it in a metastable phase (case 2).

First, we discuss the case where the cycle starts with the material in the globally ergodic equilibrium phase *A*. At some point (T,p) along the trajectory in thermodynamic space, we find that the system can/does switch into a different phase *B*—which can be either the new equilibrium phase or a metastable phase—since at and beyond the point (T,p), *A* has become metastable/unstable on the time scale of observation, $t_{obs}\geq \tau_{esc}\left(A;T,p;b_{esc}\right)$. Here, $t_{obs}$ can refer to the total time the system spends in the leg of the path in (T,p) space where *A* is only metastable at best as well as to the time allocated to a given point in (T,p) space, i.e., the point where the phase transition takes place or should take place. As far as the possible transition from phase *A* to phase *B* is concerned, we are again dealing with the balance in the time scales for the nucleation and growth process discussed in the two previous subsections, that is, the time available at any given point along the trajectory and the total time spent in the leg where phase *A* is metastable. Let us now assume that $t_{obs}\gg \tau_{esc}\left(A;T,p;b_{esc}\right)$ for this leg; then, the system will be in phase *B* starting at some point along this leg of the cycle. Of course, we again have to optimize the time allocation to this leg as a whole, and in particular to the transition stage from *A* to *B*.

At the end of this portion of the cycle in thermodynamic space, phase *B* becomes metastable and phase *A* is again the equilibrium phase. However, we note that *B* does not necessarily transform nicely back to *A*; the system might evolve into competing phases analogous to the crystallization of alternative modifications from the glassy state or an amorphous precursor mentioned in Section 3.1.2. Instead, several alternatives can occur. First, *B* may be a long-lived metastable phase $\tau_{esc}\left(B;T,p;b_{esc}\right)\gg t_{total}$ for the (T,p) values of the rest of the cycle; in that case, we will only reach *A* with a low probability on the order of $\left(b_{esc}\frac{t_{total}}{\tau_{esc}\left(B;T,p;b_{esc}\right)}\right)$. Alternatively, *B* could transform into another metastable phase *C* (with a certain probability ranging from very small to 100 %), which is a long-lived metastable competitor to phase *A*. Finally, some kind of glassy or amorphous state might appear, as discussed above in Section 3.1.2. Fine-tuning the path and the time spent in various regions of (T,p)-space along the cycle trajectory might be required in order to avoid either of these three outcomes if we want to return the working fluid to the starting phase *A* at the end of the cycle.

Second, we start in a metastable state *C* (perhaps because its properties are just perfect for our purposes). Now, we want to return to this state after finishing the cycle. As long as we spend less time at each point (T,p) on the path than the escape time $\tau_{esc}\left(C;p,T\right)$, we might be able to perform the cycle avoiding any phase transition and return to *C* at the end of the cycle. However, running through the dangerous parts of the cycle—where *C* is highly unstable—at high speed will presumably extract a considerable price in excess entropy production and lost work compared to the ideal values if we did not have to follow this fast schedule. However, if we accept the transition to a different phase *B* at some point along the cycle, we are again faced with the four possible outcomes upon return to the starting point of the cycle in thermodynamic space: we remain in the new metastable phase *B*, end up in the equilibrium phase *A*, reach the desired metastable phase *C*, or are stuck in a glassy state. Considering that at least some of the undesired options can occur with a non-vanishing probability, it appears that again a probabilistic approach to the optimization problem of (a set of) cycles will be needed (see Section 3.1.5).

**Figure 5:**
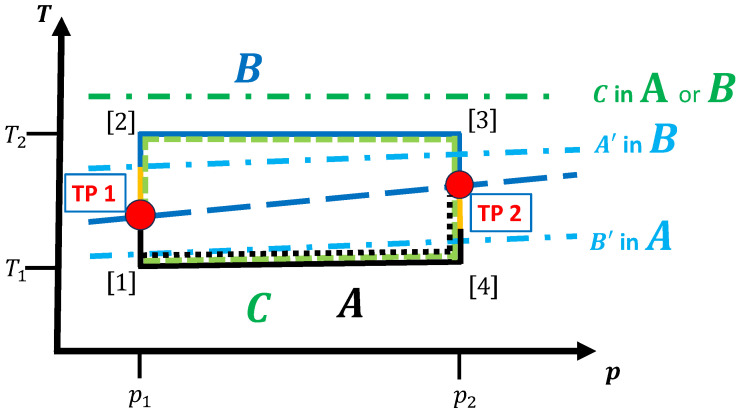
Sketch of a simple schematic path in (T,p) space involving three phases. *A* is the global equilibration phase below the dark blue line (large dashes) and *B* is the equilibrium phase above the dark blue line (large dashes), while the blue (dash-dotted) lines indicate the range of metastability of phases *A* and *B* in the part of the thermodynamic space where the equilibrium phases are *B* and *A*, respectively. The dark green (dash-dotted) line indicates the range up to which the phase *C* is metastable; note that *C* is never the equilibrium phase. TP1 and TP2 indicate the points along the thermodynamic path the system is following, at which the equilibrium (infinite-time) phase transitions between *A* and *B* would occur. The colors along the path in (T,p)-space indicate which of the phases are present: black = equilibrium phase *A* is present at the start and after nucleation from the phase *B* (after *B* has become metastable); orange = metastable phase *A* or metastable phase *B* is present before the transformation to the equilibrium phase takes place (as in Figure 3); dark blue = equilibrium phase *B* is present; dashed green = metastable phase *C* is present; and dotted black = equilibrium phase *A* is present after nucleation from the metastable phase *C*.

Figure 5 shows how the possibility of several outcomes when dealing with phase transitions in finite times may result in different (meta)stable phases along the path in thermodynamic space. Starting with the equilibrium phase *A* at point [1], two possible transitions can occur at the phase transition point TP1. First, phase *A* can continue while being metastable until transforming into the new equilibrium phase *B* where the system now remains until the phase transition point TP2 is reached; at that point, phase *B* becomes metastable and subsequently transforms either into the equilibrium phase *A* (again after some delay) or the metastable phase *C*, and it is assumed that no glassy state is present in the system. Alternatively, at TP1 we can switch into the metastable phase *C* and remain in this phase until TP2 is reached; there, we either stay in the metastable phase *C* until we reach the starting point [1] again, or we transform back into the equilibrium phase *A*. Of course, there are other possibilities, such as the system staying in phase *B* (now metastable) until the end. Keeping track of these possible bifurcations results in a decision tree, as discussed in Section 3.1.5.

We recall that the material can enter in several ways when employed as a working fluid, and can influence the analysis of the thermodynamic cycles. From a modeling point of view, this can pose an external source of problems that, for one, impose restrictions on the speed along the path or the choice of path as such. For example, one might like to avoid the unstable region *C* at the end of the cycle (case 1 above), or perhaps not want to transform the working fluid into phase *B* at all if possible. To achieve this, one could, e.g., take a worse (e.g., more inefficient) path in (T,p)-space, e.g., stay below the phase transition line that separates phases *A* and *B* as far as the process is concerned, while still performing the desired work, or alternatively by using a different range of temperatures that has the disadvantage of producing less work than one would like or generating more excess entropy. If this avoids the large amount of extra thermodynamic work needed to recreate the original material, it might be worth the thermodynamic price. Here, we note that if the transformation to phase *B* is an integral part of the functioning of the thermodynamic engine, then it of course cannot be excluded from the thermodynamic cycle.

As we have seen already, a second issue is that the choice of material can impose an additional fixed cost, e.g., of transforming the material back to *A* from *C*. This might occur in case 1 discussed above, and the extra cost needs to be added on top of the otherwise optimized route. We note that the phase transition A→B is assumed to be already included in the optimization procedure, since it belongs to the “ideal” cycle we are planning to follow.

A third concern might be that we can, e.g., achieve certain goals more easily when the material has the properties of modification *B* while running the engine, but phase *B* is environmentally unstable (susceptible to rusting) or unsafe (poisonous), and as such should be transformed back to *A* after the process is finished and the engine is at rest. An example might be a switch from a stable insulating material *A* to an unstable but conducting material *B*, where the electrical conductivity is important for the performance of the cycle, e.g., in a battery.

#### 3.1.4. Challenges of Cyclic Processes

A number of the issues discussed in the previous examples apply to both cyclic and line-type processes (where the starting point and end point of the path are different, and the path does not intersect itself), since any phase transition with inconclusive outcomes will require the introduction of a probabilistic setup for the optimization of the finite-time process. Similarly, any phase transition that is encountered along the path will introduce possibly large non-equilibrium states of the system. Thus, it becomes problematic to use the straightforward intuitive (linear response type) models of the relaxation to approximate equilibrium, which underlie our ability to perform, e.g., analytical calculations when optimizing the finite-time process. These aspects are present for both cyclic and line-type paths in thermodynamic space. Cyclic processes employing real materials instead of abstract thermodynamically perfect ones exhibit their own challenges when compared with a given line-type process in thermodynamic space, even if the line-type process also involves various kinds of phase transitions. As pointed out earlier, the major issue for cyclic paths is the ability to return to the original starting point with the properties of the material that serves as the working fluid being intact and identical to the state at the beginning of the cycle.

Several detrimental outcomes have already been discussed, such as the material being in a different (meta)stable phase when returning to the starting point of the cycle in thermodynamic space. Examples we have considered are a metastable phase *B* or *C* instead of the original equilibrium phase *A*, the equilibrium phase *A* instead of the original metastable phase *C*, the metastable phase *B* instead of the metastable phase *C*, or some kind of glassy state. We have also noted that if we were to start with the material in a glassy state, then aging processes during the cycle can result in the material being in a slightly different aged (or rejuvenated) glassy state when returning to the starting point at the end of the cycle, since the glassy material is not in a locally ergodic equilibrium state but in an ever-evolving marginally ergodic quasi-equilibrium state.

In fact, this issue, raised by the somewhat ill-defined glassy state where the material is permanently in a slowly evolving quasi-equilibrium, can also appear when employing metastable and thermodynamically stable crystalline modifications of the material as a working fluid. The reason is that in these systems we are often dealing with the accumulation of defects in each cycle, which are not eliminated or brought back to their equilibrium concentrations and spatial distributions after the cycle has ended: the material is back in its original modification (or has never left it), but many long-lived defects have been created while undergoing the cyclic thermodynamic process. If these defects affect the physical or chemical properties of the working fluid, then subsequent cycles will yield slightly different outcomes even for the same path in thermodynamic space with the same allocation of time along this path. Of particular importance are long-lasting non-equilibrium defects on the atomic or mesoscopic level, such as defect clusters, dislocations, domain boundaries, or grain boundaries. In the last case, we have usually already crossed the line to essentially permanent changes on most time scales and for most systems of interest, since now we are dealing with a polycrystalline material instead of the single-crystal material in thermodynamic equilibrium that we started with.

Here, we remark that sometimes these non-equilibrium states are preferable as working fluids because of their specific macroscopic properties. For example, if a glass is replaced by a poly- or single-crystalline material as the working fluid, it might no longer have the desired property spectrum, e.g., it might be more brittle, less transparent, etc. In addition to glasses, an extreme case of such materials with a desirable high “defect” density on the atomic level might be so-called “high-entropy” materials [140,141], where we aim for a state with a high degree of so-called “controlled disorder” [7], such as a super-sized version of a solid solution. As mentioned earlier, solid solutions are equilibrium or long-lived near-equilibrium states; often, a material called a “solid solution” is not yet fully relaxed into thermodynamic equilibrium, and “emits”/“absorbs” heat while settling into the maximum entropy state. However, these high-entropy solid (solutions) do not necessarily correspond (and actually are unlikely to correspond) to thermodynamic equilibrium phases/states at the temperatures and pressures at which the material is being used in devices. As a consequence, (T,p) cycles performed using such a material as a working fluid can very well lead to a change in structure (on the atomic level and on the mesoscopic level), in particular regarding the compositional atom distribution throughout the material, and properties of the material can even be different after the cycle. Another example for a type of quasi-equilibrium material where thermodynamic cycles (in finite time) are important regarding its properties are battery materials, since each cycle adds a certain number of more or less permanent “defects”. Here, each discharge–recharge cycle (at constant temperature and pressure but at different external applied voltages) leaves the material slightly changed (in a thermodynamically lower metastable state), until no further cycles are possible and the battery needs to be replaced and the material recycled.

The accumulation of defects raises issues that are also present when we consider the finite size of the system or when we are dealing with an inhomogeneous material. In order for an inhomogeneous material to be the equilibrium phase, the material usually needs to be a composite material or agglomeration, otherwise the system will slowly evolve towards a homogeneous new (!) material. If we are dealing with such a composite, then a contact boundary is present, with its own physics and chemistry; in particular, we encounter possible mixing on the atomic level via diffusion of atoms between the phases in the boundary zone. At finite times, we cannot achieve a smooth distribution of the “foreign” atoms as minority “defect”/“solid solution”-type atoms inside the other phase. Thus, in each of the separate phases there is a gradient of the minority atoms in the “other” phase, violating the condition of homogeneity in that subset of the composite material. The equilibrium amount of minority atoms depends on the (T,p) values of the phase diagram; thus, at least locally in the interface zone, we deal with changes in this concentration as function of (T,p). Further “irreversibilities” can appear in the quasi-permanence of cracks and grain boundaries, which are typical mesoscopic features of a material.

This brings up the issue of the finite size of the material employed as a working fluid: if we are down to mesoscopic sizes, e.g., employing single crystalline grains of a material, then this might allow the system to fully equilibrate without appealing to the self-averaging principle. In particular, we could have single crystal-to-single crystal transformations, i.e., we do not end up with some of the essentially irreversible changes mentioned earlier, such as crystal-to-powder-like structure changes on the mesoscopic level, which destroy the macroscopic reversibility and prohibit the return of the material to its state at the beginning, thereby violating the assumption underlying every thermodynamic cycle. Another basic advantage of mesoscopic-size working fluids is that we do not need to worry too much about the speed with which external changes in the thermodynamic conditions propagate and spread through the material serving as the working fluid. On the other hand, the size of the surface is quite large compared to the volume of the material; thus, surface terms should be taken into account when computing thermodynamic functions for the system. Furthermore, every phase becomes metastable for mesoscopic size materials—including the thermodynamically stable one, in principle—and the number of metastable phases in the system can increase considerably. Finally, many thermodynamic engines must employ working fluids of macroscopic size in order to be useful in real-life situations.

#### 3.1.5. Probabilistic Optimizations

In the previous subsections, we have already encountered one general way to deal with those cycles where the system ends up with a final material that is different from the original one. First, it is necessary to add a certain amount of work after the actual work cycle has been performed in order to rejuvenate the material and transform it back to its original state. This work, and in principle the time required to perform this work, needs to be taken into account when formulating the finite-time optimization problem, e.g., by subtracting the time needed for restoring the original phase $t_{rejuv}$ from the total time $t_{total}$ available for the cycle, such that we can start the next cycle directly after the material is restored.

As mentioned in the previous subsections, when dealing with competing metastable phases, one obtains a probability (as function of temperature and pressure) that indicates the likelihood of the metastable phase *i* leaving its locally ergodic region $R_{i}$ on the energy landscape within the observation time spent at this point in thermodynamic space. In the definition of the escape time, this probability is provided in the form of the characteristic parameter $b_{esc}$, which indicates that an atomistic trajectory of length $\tau_{esc}$ will leave the region $R_{i}$ with a probability exceeding $b_{esc}$. In the definition of the escape time, $b_{esc}$ is usually assumed to be very small, such that even a small likelihood of leaving the system counts for defining the time scale $\tau_{esc}\left(i;T,p;b_{esc}\right)$. Conversely, for a given time scale of interest $t_{obs}$ which we want to spend at the point (T,p), we can set this time equal to the escape time, allowing us to compute the corresponding probability $b_{esc}$. As a consequence, once in every $1/b_{esc}$ cycles on average, the system will end up leaving phase *i* and end up in the correct (if we wanted to leave phase *i*) or incorrect (if we wanted to stay in phase *i*) phase.

Now, we can construct a “decision”-like tree of outcomes for the cycle as far as the phases that occur during the trajectory in thermodynamic space are concerned, where each decision (i.e., whether a transition occurs or not along the path) is noted. For each transition that takes place, we can include the times needed for the transition in the list of legs of the path and also add the amount of excess entropy production/loss of work associated with this transition to the cost function of the finite-time optimization problem. For each path on the decision tree, we can compute the probability that the system will follow this decision tree path. Furthermore, we can treat each such path of the decision tree as a separate finite-time optimization problem that needs to be analyzed, taking into account the constraints due to the transitions that are defined to occur.

In the next step, we optimize the time allocation/distribution for every given path on the decision tree with respect to the thermodynamic cost function of the cycle. Here, we must decide whether, for a decision path where the “wrong” state of the material is found after the regular thermodynamic cycle has been finished, we are willing to “rejuvenate” the material to its original phase as part of the cycle or not. In the former case, we need to subtract this rejuvenation time $t_{rejuv}$ from $t_{total}$ and add the associated thermodynamic cost to the cost associated with this decision tree path. Alternatively, we could instead “replace” the material that ended up in the undesired phase with the material in the correct original phase obtained from a large reservoir/storage, such as that provided by, e.g., a recycling company. In the latter case, it is not quite clear which cost and time should be associated with such a replacement; a compromise might be that we do not subtract the replacement time from $t_{total}$—after all, the recycling company will rejuvenate enormous amounts of spent material in one fell swoop—yet still add the thermodynamic work needed in this external recycling process to this path.

Furthermore, we note that when optimizing the cycle following such a decision tree path we might end up influencing the occurrence probability of this path by varying the time we spend within the region in thermodynamic space where this transition occurs. This kind of feedback should be automatically included in the complete optimization problem until a self-consistent solution has been reached. In this discussion, we assume that the probabilities of the transitions occurring in the first place do not change by very much when we optimize the cycle for a given sequence of transitions assumed to occur along this decision tree path.

In a second step, we would now consider an ensemble of cycles representing all the decision tree paths and assign their appropriate probabilities. Because these probabilities will change depending on the choice of path in thermodynamic (T,p)-space, we can now perform an optimization on the path the cycle should traverse in thermodynamic space. Of course, for each choice of cycle path we need to perform the time allocation optimizations for all the decision tree paths together with a re-determination of the probabilities with which such decision tree paths will occur.

### 3.2. Optimal Schedule of Processes Aimed at Synthesis/Production of Materials

In the previous Section 3.1, we have discussed many aspects of thermodynamic cycles in finite time that involve a working fluid with a complex energy landscape where multiple locally ergodic and/or glassy regions are present on the energy landscape for the area of thermodynamic (T,p)-space in which the process takes place. The typical targets of the optimization are, e.g., minimization of the excess entropy/heat production or loss of work/generation of extra work due to the finite time available for completion of the cycle. Another important class of optimization problems associated with thermodynamic processes following some trajectory in thermodynamic space consists of the production of chemical compounds and materials in finite time. Here, the goal is the maximization of the desired product, where the target compound does not necessarily constitute the thermodynamic equilibrium phase of the system. Alternatively, for a given amount of product, the goal might be to minimize the amount of work or heat production if the product needs to be generated within a certain finite time.

Of course, the efficient production of chemicals has a long tradition in chemistry and chemical engineering, with many processes having been analyzed and optimized in the past [142,143,144,145]. We also find examples where such analyses have been performed by employing the viewpoint of finite-time thermodynamics, such as studies of distillation processes [146,147], various chemical reactions [148,149], or maximization of the desired product obtained upon cooling from a melt by a nucleation and growth process [128]. However, such analyses have usually not taken the complex energy landscape of the underlying chemical system into account.

Designing syntheses that target specific molecules that would never form by themselves from a given initial set of atoms or small molecules have been highly successful [150,151,152], with molecular chemists developing a plethora of so-called elementary name reactions [153] that describe specific reaction steps in the multi-step reaction paths starting from very simple and widely available educt molecules. We note that many of these target molecules correspond to high-lying minima on the energy landscape of the chemical system that is defined by the set of atoms making up the molecule. Due to the high degree of kinetic control of the chemical reactions involved in building up these specific molecules, moving from one minimum to the next on the energy landscape can be done in a controlled fashion.

In contrast, aiming for the synthesis of specific solid phases and designing efficient synthesis routes for this purpose is a much greater challenge for the experimentalist, even if the goal is only to obtain the thermodynamically stable phase [154,155]. One problem is that for many compositions in a chemical system even the equilibrium phase is not known with certainty. Trying to solve the task of identifying all stable solid phases of interest in a chemical system spawned the field of crystal structure prediction over thirty years ago (for reviews, see, e.g., [154,156,157]), which is now in the process of adding machine learning to its toolbox [158,159]. However, the major reason for the difficulty in systematically synthesizing such metastable solid compounds in the experiment is probably the lack of atom-level kinetic control of the chemical processes. Instead, thermodynamics-based tools such as variations of temperature, pressure, and attempts to (locally) vary the concentration of starting atoms or precursor materials (crystalline, amorphous, or layers of films) are mainly employed. Trying to alleviate this issue has led to various film- or atom layer-based methods [134,160,161], where systematic quantitative analyses of the outcome of the growth of various crystalline phases (e.g., from an amorphous precursor) have been performed as function of the applied (thermodynamic parameter-based) synthesis schedule [135,136]. However, there is clearly still a long way to go in this regard.

From the optimization point of view, the major challenge is to guide the system into the right metastable target phase within finite time, where we assume that we have full information about the set of relevant local minima and locally ergodic regions on the energy landscape together with information about the generalized barrier structure including both energetic and entropic barriers. The issues that arise are in many ways quite analogous to those discussed in Section 3.1: the many time scales associated with the movement on the energy landscape when exiting from or equilibrating in (intermediary) metastable phases or amorphous precursors, the amount of excess entropy/loss of work when trying to accelerate nucleation and growth processes, the persistence of quasi-equilibrium states such as glasses, and the probabilistic nature of the outcome of the synthesis even for identical schedules in (T,p)-space when metastable phases are targeted or need to be passed through on the route to the final solid compound of interest.

To address these issues, it is possible to employ empirical theoretical models for the various stages of the envisioned synthesis route. For example, the maximization of the amount of a crystalline solid phase generated via cooling from the melt within a finite time $t_{total}$ has been investigated [128], where the temperature served as the thermodynamic control parameter. Using standard elementary models to describe the nucleation and growth stages of the process, it was found that the solution to the optimization problem consisted of a bang–bang-type schedule, with the temperature abruptly switching between a minimum value where the nucleation rate is high for the super-cooled melt and a maximum value where the growth rate of these nuclei is maximized (note that this maximum temperature must stay below the melting temperature of the material to avoid a re-melting). The same study investigated the competition between two different metastable phases, where the optimization goal was to preferentially generate one of these two phases during their growth from the super-cooled melt. Here, a bang–bang-type solution for the control parameter (in this case the temperature) was again obtained.

In the above study of crystallization from a melt, it was implicitly assumed that empirical models for both the nucleation and growth processes were available, together with values of the parameters in these models for the chemical system of interest. Furthermore, it was assumed that all relevant metastable phases in the system were known, such that the predictions would be able to guide the experimentalist in their syntheses. However, for many systems no such experiments have yet been performed, or none of the metastable phases that compete with the known equilibrium phase have yet been synthesized; thus, the empirical laws guiding the processes involved are not available to perform optimization with.

While distressing on the one hand, on the other it is clear that even some qualitative guidance in thermodynamic space would already be of great help for the experimentalist in finding ways to synthesize any of the metastable solid phases that have been predicted or intuited to exist in the system, thereby spurring theoretical work in the field even using very rough approximations. To address this challenge of synthesis route prediction, it is necessary to perform the finite-time optimization directly on the energy landscape of the system of interest [7]. A prerequisite is detailed knowledge of the structure of this landscape, including all local minima of relevance that individually (in the case of crystalline modifications) or in structurally related groups (in the case of solid solution phases) are at the center of locally ergodic regions corresponding to these (meta)stable phases, the (effective) local densities of states for these minima and associated locally ergodic regions, and the energetic and entropic barriers separating these regions. These generalized barriers are captured in form of probability flows between the locally ergodic regions as function of energy during the global landscape explorations [21,94,109]. Such flows, together with the local densities of states, can be determined using the so-called threshold algorithm [38,124,162], which explores the regions of the energy landscape which are accessible from all minima of relevance below a sequence of energy lids.

This information can be used to construct a Markov model description of the dynamics of the system on the level of the locally ergodic regions [7,163], i.e., we model the probabilistic dynamics on the energy landscape in a coarsened picture that considers the movement of the walker between locally ergodic regions instead of from microstate to microstate, as would be done in, e.g., a molecular dynamics simulation. The thermodynamic parameters such as temperature and pressure influence the transition probability entries in these Markov matrices via the Boltzmann factors and shifts in the enthalpy levels; we recall that a change in pressure from $p_{1}$ to $p_{2}$ corresponds to a shift $(p_{2}-p_{1})V$ in the potential enthalpy of the microstates of the system. As a result of the finite-time optimization, we find an optimal temperature–pressure schedule in thermodynamic space for each target phase; these schedules can then serve as a rough guideline for experimentalist to design their synthesis routes.

Examples of such thermodynamic optimizations on the energy landscape level include studies by Hoffmann et al. [163,164,165], who employed the results of energy landscape investigations of periodic approximants of the magnesium difluoride system [94,166,167] to construct such Markov matrices for the probability flows as function of temperature and pressure. Starting the Markovian time evolution from the system at very high temperatures, the optimal temperature-pressure schedules are computed, enabling to obtain not only the experimentally-known rutile structure but also predicted metastable alternative phases such as the anatase and the CdI_2_ modification to be obtained with a certain probability.

Such in-principle studies constitute only the beginning of the applicability of such synthesis optimization to realistic systems. The main problem when constructing simulations on the level of the metagraph of the locally ergodic regions for solid state chemical systems is the number of atoms involved. Thus far, it is only possible for small periodic approximants to obtain the detailed landscape information required for the construction of the Markov model—single molecules or clusters are clearly much easier to deal with in this regard! The problem is that unless the transformation between the metastable phases occurs via, e.g., a second-order phase transition (as discussed above), real solid materials typically undergo nucleation and growth processes or grow from glassy or amorphous precursors. Thus, obtaining the appropriate time scales for the probability flows that allow us to model the Markovian evolution on the metagraph of the locally ergodic regions in a quantitatively realistic fashion requires information from landscape explorations for state spaces consisting of hundreds or thousands of atoms/variable periodic simulation cell, ideally on the ab initio level of energy. Nevertheless, such explorations are expected to become feasible with the availability of machine learning (ML) potentials for multi-atom systems, as the ML energies of these systems for multi-atom configurations in solids are reasonable approximations of the ab initio energies for the same configuration but can be computed orders of magnitude faster [168].

In this context, we comment on the issue of computing free energy differences and free energy barriers on the atomic level via molecular dynamics or Monte Carlo simulations of single walkers (or ensembles thereof) for systems that exhibit several locally ergodic regions that might compete with each other along the path in thermodynamic space. Examples of classic approaches to computing free energy differences, e.g., between two systems that can be transferred into each other by some change in characteristic parameters (such as the strength in the atom–atom interactions) or between the same system but at two different points in thermodynamic space, include the thermodynamic integration method and the thermodynamic perturbation method [169,170]. The basic assumption behind such approaches is the observation that the work needed to perform such a transformation/movement on the energy landscape constitutes a lower or upper bound on the free energy difference [171]. The closer the system can stay to (local) equilibrium during the procedure, the tighter these bounds will be. Usually, the transformation is performed in both the forward and backward direction, at least as long as we are moving between two global equilibrium states. The same considerations apply when the transfer is supposed to take place between two (meta)stable modifications *A* and *B* at the same location or at different locations in thermodynamic space.

In a practical realization of such a computation, one would employ an ensemble of walkers on the energy landscape. The movement of these walkers as the thermodynamic parameters are changed to drive the system from phase *A* to phase *B* then corresponds to a moving ensemble average along the path in thermodynamic space. If the transformation takes place in finite time $t_{total}$, either extra work needs to be expended or excess heat generated, as the system will always be slightly out of equilibrium. As a consequence, we are again dealing with a finite-time optimization problem, i.e., attempting to find the optimal path in thermodynamic space to move from phase *A* to phase *B* while keeping the ensemble representing the system close to equilibrium everywhere along the path. In addition, we need to identify the allocation of the available time along the path.

The general question of optimally moving a system in thermodynamic space on the level of the energy landscape, and thereby computing the free energy differences, has been addressed in the literature [30]; however, those derivations assume that no bifurcations will be encountered. If other locally ergodic regions or marginally ergodic regions—corresponding to metastable phases or glassy solids, respectively—can be accessed along with the target phase *B* with non-vanishing probability, then the ensemble will no longer stay in or close to thermodynamic equilibrium, since some of the walkers will visit or even end up in other locally ergodic or marginally ergodic regions of the landscape. Ensuring that all walkers reach phase *B* requires additional work performed on the system, adding to the uncertainty in the free energy calculation already present due to the finite-time effects along the “correct” route through the energy landscape.

In addition to accepting this handicap and paying the price of extra work or entropy/heat production to force the walkers to stay on the direct route between phases *A* and *B*, one can in principle again employ a decision-tree approach, as mentioned in Section 3.1.5, where a probabilistic formulation of the many possible outcomes of the cyclic thermodynamic paths was presented. The advantage of such an approach is that one does not add essentially uncontrollable forcing terms into the algorithm; furthermore, as a positive side-benefit, estimates for free energy differences among many metastable phases of the system can be obtained. The disadvantage is the enormous number of walkers needed to probe the energy landscape in a locally equilibrated way along many possible pathways through the landscape between the locally ergodic regions. The possible appearance of glassy states is another serious handicap of an unbiased approach, since such non- or at best quasi-equilibrium states might not be exited on realistic simulation time scales, possibly requiring the system to be heated close to the melting point. Such a deviation from the original thermodynamic path will quite likely result in many additional uncertainties in the calculation of the free energy. Nevertheless, addressing such issues is an important task in the context of free energy computations for systems with complex energy landscapes.

### 3.3. Systems with Complex Energy Landscapes Outside Physics and Chemistry

Systems with complex energy landscapes are also found outside the fields of chemistry and physics, ranging from mathematics and biology over engineering and economics to the humanities; for an overview, we refer to [8]. In the latter cases, the high-dimensional single or vector-valued function over a large space of microstates is often no longer called an energy function; instead, we speak of a generalized cost function. To provide some specific examples, this function is called the fitness function (which is to be maximized) in biological systems when discussing evolution; the welfare function in the context of thermo-economics for multi-agent systems; the happiness function for social systems; the cost function for planning problems in business level economics; and the objective function in abstract or practical combinatorial and global optimization problems in mathematics.

In many of these systems, one is mostly interested in identifying the local and global minima and maxima of the generalized cost function, i.e., most of the effort is devoted to developing or applying suitable global optimization techniques and algorithms. Because these algorithms must explore the landscape in an efficient fashion, many of them are inspired by the way in which physical and chemical systems proceed in a natural way to explore their energy landscapes in order to reach thermodynamically stable phases. Examples of such algorithms are the simulated annealing method [172] and genetic and evolutionary algorithms [173], which have spawned a multitude of variants. Here, the picture drawn from classical mechanics of a system rolling downhill under the force of gravity to reach a state of lower potential energy leads to the deterministic gradient descent approach, while stochastic methods involving random walks on the cost function landscape reflect the statistical nature of the approach to low-energy minima associated with equilibrium phases in statistical thermodynamics. Such algorithms have been analyzed by employing the analogy of a glass transition or a glassy intermediary region on the energy landscape, which must be passed through before the desired low-energy cost function minima can be identified [174,175].

The challenges faced in the design and optimization of global optimization algorithms to explore such multi-minima cost function landscapes with a limited amount of computational resources are very similar to those encountered in thermodynamic space when moving from a phase that is stable at high temperatures to the thermodynamically stable equilibrium phase at low temperatures discussed above. In this context, we note that the energy landscapes of such combinatorial optimization problems frequently do not exhibit a well-separated ground state basin that would be analogous to the well-defined crystalline thermodynamic equilibrium phase at low temperatures. Instead, many of the landscapes of such optimization problems are more similar to those of spin glasses, which by definition or construction do not have a well-defined locally ergodic region surrounding the global energy minimum. Instead, many minima with nearly the same energy as the global minimum energy exist on the complex energy landscape, and these are located far away from the global minimum. This constitutes a qualitative difference from the energy landscape of a crystalline material modeled with a realistically large periodic approximant such that isolated defect configurations can be included; in the latter case, all low-energy minima that have nearly the same energy as the global minimum correspond to equilibrium defect configurations of the thermodynamically stable zero temperature equilibrium phase, and as such belong to the same locally ergodic region.

Quite generally, employing a whole ensemble of walkers on the cost function landscape as opposed to only a single walker allows us to compare the evolution of a thermodynamic system toward a (meta)stable phase with the gradual establishment of (meta)stable states of biological, ecological, social, or economic systems, which can reach an equilibrium-like state regarding the exchange of (biological or economic) goods and resources with a hypothetical external environment. In particular, when moving from one metastable biological, ecological, or economic state to another, we encounter problems similar to those we have discussed for the movement between two metastable solid phases: we need to invest a large amount of “work” or resources to accomplish the transformation into the desired biological, ecological, or economic target state. Doing this while minimizing the extra work or loss of resources within a finite time is clearly analogous to a finite-time optimization problem. We also note that chaotic and nearly unpredictable changes between two stable biological, ecological, or economic states can occur in such systems. This can result in the system being in a non-equilibrium or quasi-equilibrium situation which can persist for very long times, in analogy to the glassy states of chemical materials.

More concretely, in biological systems we can consider scenarios such as the attempt to breed certain traits into farm animals or to change the resistance of plants against “pests” within as few generations as possible or while employing a minimal number of intermediary breeding animals to be analogues of the scenario involving transformation of a given solid phase into a different metastable material in an as efficient a manner as possible. Similarly, we can consider the recovery of an ecosystem [176,177] after, e.g., a destructive volcanic eruption. Typically, the ecosystem observed in the region *V* around the volcano must proceed through a series of metastable ecosystems featuring pioneering and other intermediary plant and animal generations before a stable ecosystem is reestablished. Achieving this with a minimal amount of effort within a finite time, perhaps measured in few decades, requires careful fine-tuning of the environmental conditions experienced by the region *V* as function of time, which can strongly influence the types of plants and animals that will grow and settle in region *V* after the disturbance. This recovery process is quite analogous to a series of phase transitions when moving from, e.g., the melt to the low-temperature equilibrium phase via several intermediary (high-temperature) phases after the system has experienced an abrupt change in its thermodynamic environment, such as a quench in temperature and/or exposure to a cycle of high and low pressures.

Such environmental boundary conditions can include the general climate of the region, the plants or animals introduced by humans in region *V* after the volcanic eruption, and/or the (fixed) distribution of plants and animals in the geographic region *G* surrounding the region of interest near the volcano *V*. While the local climate or weather are difficult to influence by human intervention, the plant and animal populations in the surrounding region *G* can be controlled by humans. For example, if region *G* exhibits an agricultural monoculture or if predatory animals (wolves, bears, etc.) are systematically eliminated in *G*, then this will have a different influence on the final ecological state in region *V* compared to if region *G* were a wild forest. Note that the ecosystem reached in the long-time limit may be different from the original one before the volcano erupted; there can be many feasible metastable ecosystems that are stable on long time scales in region *V*, and the one the system settles into might depend on the environmental boundary conditions. We remark that there is actually no reason to assume that these boundary conditions completely determine the final ecosystem in region V; in principle, many long-time metastable ecosystems could exist for the same set of environmental boundary conditions.

While it is clear that there are many fascinating examples of (thermodynamic-like) processes for systems that have complex energy landscapes in fields of science outside the realm of physics and chemistry, we do not want to go into greater detail here as far as these biological, ecological, social, and economic systems are concerned. Considering, e.g., the mathematical formulation of thermo-economics and expounding the correspondence of its variables with those of thermodynamics would require a lengthy presentation which is beyond the purview of this perspective. Nevertheless, it should be clear that the concepts of finite-time thermodynamics are applicable to many of these non-physical systems, and can provide guidance about the optimal route toward the establishment of the desired states in these systems. Conversely, insights gained from dealing with systems in biology, ecology, or economics may inspire new work in the finite-time thermodynamics of chemical and physical systems.

## 4. Conclusions

After a short presentation of some fundamental concepts of finite-time thermodynamics and complex energy landscapes, we have discussed a number of prototypical processes and cycles that involve phase transitions of the working fluid material or the target material of a synthesis process. A major focus has been on cycles that include a simple back-and-forth transition between two equilibrium phases, at least in the limit of infinite time available for performing the cycle. To study such systems in the context of finite-time thermodynamics, it is necessary to employ appropriate models for the nucleation and growth processes involved in the phase transitions, in addition to models describing the relaxation to equilibrium along those parts of the thermodynamic path where the system stays within an equilibrium phase. Similar considerations arise when we try to optimize synthesis routes to achieve specific (meta)stable phases in a given chemical system, where the synthesis includes standard first-order phase transitions or where various nucleation and growth processes occur, starting from thermodynamically stable materials and also from those that are not in thermodynamic equilibrium, such as amorphous precursors or glasses.

In principle, this task is straightforward as long as the available time for the whole process $t_{total}$ is sufficiently large to allow the system to remain close to equilibrium along the whole path in thermodynamic space and no bifurcations occur. In that case, the main challenge is to adapt and derive appropriate models for the above mentioned relaxations and transitions, and then to implement them as part of the optimization problem.

However, when analyzing the time scales involved, in particular those of the nucleation and growth processes during the phase transitions, the equilibration and escape times associated with the many competing metastable phases, and the time scale on which glassy materials very slowly convert into the crystalline equilibrium phase, it becomes clear that such cycles can run into major problems in practical applications. The reason for this is that the finite time $t_{total}$ available for finishing the thermodynamic work cycle or line-type path is often not sufficient to guarantee that the material employed as the working fluid is transformed into precisely the specified phase or state via the planned phase transitions along the path. In addition to encountering difficulties in reaching the intended phases during the process itself, the system might become stranded in alternative (meta)stable phases or a glassy state at the end of the process, i.e., the working fluid might not be returned to its original thermodynamic and structural state upon return to the origin of the cycle in thermodynamic space or the desired product material might not be obtained in sufficient amounts or purity. As a consequence, for a non-negligible fraction of the cycles, large amounts of undesired (metastable) phases or glassy states can appear along the thermodynamic path or non-equilibrium defects may accumulate in an essentially irreversible fashion.

Performing (and optimizing) these processes will presumably require quite radical measures; a large amount of additional work or time $t_{rejuv}$ may have to be invested after the end of the cycle in order to rejuvenate the material back into its original state, especially if one wants to repeat the cycle many times as part of a long-time process, in which case it is not usually possible to discard the working fluid at the end of the cycle. Essentially, one would have to add a recycling stage for the material in its final state or add a generic loss term to the final accounting of the process. Nevertheless, finite-time optimizations will be useful even here in trying to minimize the number of cycles where irreversible changes in the working fluid take place. Alternatively, it would be necessary to substantially alter the work cycle or synthesis path in thermodynamic space, avoiding all possible appearances of undesired phases and states of the material.

In trying to address this problem in full generality, we must account for all possible paths that the system might follow as far as the phases and states it can display are concerned. This requires the construction of probabilistic trees for a systematic analysis of the possible bifurcations during the thermodynamic process under study: in this approach, we note the possible outcomes together with their probability of occurrence at each point or leg along the path in thermodynamic phase space where a transition to a (meta)stable phase or quasi-equilibrium state such as a glass can occur. Combining this information with the models for the nucleation and growth processes and for the relaxation processes as functions of temperature and pressure, we can formulate and investigate the probabilistic optimal control problem for performing finite-time processes involving bifurcations regarding both the choice of path in thermodynamic space and the time allocation along this path.

In addition to the use of materials as working fluids that can experience phase transitions during a thermodynamic cycle in finite time or designing efficient synthesis routes towards new materials that exhibit metastable phases, there exist a multitude of other systems that also exhibit complex energy landscapes with many long-time stable states, analogous to the metastable phases in chemical systems; such systems appear in economics, where many different business strategies and economic systems can be stable on reasonably long time scales, as well as in biology and ecology, where many different kinds of animals and plants can survive in similar ecological niches and where whole ecosystems can be realized for the same external geographical and climate conditions. Studying and optimizing the dynamics of these systems that exhibit stable states which can be transformed into alternative ones—but possibly with high costs if the transition is forced to take place on short time scales—can potentially benefit from the use of methods developed for the study of finite-time thermodynamics and the optimization of thermodynamic cycles in finite time.

## Data Availability

Data is contained within the article.

## References

[1] Tisza L. (1966). Generalized Thermodynamics.

[2] DeHoff R.T. (1993). Thermodynamics in Materials Science.

[3] Swendsen R.H. (2012). An Introduction to Statistical Mechanics and Thermodynamics.

[4] Goldstein H. (1978). Klassische Mechanik.

[5] Simonyi K. (2004). Kulturgeschichte der Physik.

[6] Lautrup B. (2005). Physics of Continuous Matter.

[7] Schön J.C., Reedijk J., Poeppelmeier K. (2023). Energy landscapes in inorganic chemistry. Comprehensive Inorganic Chemistry III.

[8] Schön J.C. (2024). Energy landscapes—Past, present, and future: A perspective. J. Chem. Phys..

[9] Pathria R.K. (1996). Statistical Mechanics.

[10] Sieniutycz S., Salamon P. (1990). Finite Time Thermodynamics and Thermoeconomics.

[11] Bejan A. (1996). Entropy generation minimization: The new thermodynamics of finite-size devices and finite-time processes. J. Appl. Phys..

[12] Hoffmann K.H., Burzler J.M., Schubert S. (1997). Endoreversible Thermodynamics. J. Non-Equil. Thermodyn..

[13] Andresen B. (2011). Current trends in Finite-Time Thermodynamics. Angew. Chem. Int. Ed. Engl..

[14] Curzon F.L., Ahlborn B. (1975). Efficiency of a Carnot engine at maximum power output. Amer. J. Phys..

[15] Andresen B., Berry R.S., Nitzan A., Salamon P. (1977). On lumped models for thermodynamic properties of simulated annealing problems. Phys. Rev. A.

[16] Reitlinger H.B. (1929). Sur l’Utilisation de la Chaleur dans le Machine de Feu.

[17] Novikov I.I. (1958). The efficiency of atomic power stations. J. Nucl. Energy.

[18] Bejan A. (1982). Entropy Generation through Heat and Fluid Flow.

[19] Sciubba E. (2001). Beyond thermoeconomics? The concept of extended exergy accounting and its application to the analysis and design of thermal systems. Exergy Int. J..

[20] Schön J.C., Jansen M. (2001). Determination, Prediction, and Understanding of Structures Using the Energy Landscape Approach—Part I. Z. Krist..

[21] Schön J.C., Wevers M.A.C., Jansen M. (2003). Entropically stabilized region on the energy landscape of an ionic solid. J. Phys. Cond. Matter.

[22] Farquhar I.E. (1964). Ergodic Theory in Statistical Mechanics.

[23] Palmer R. (1982). Broken ergodicity. Adv. Phys..

[24] Thirumalai D., Mountain R.D., Kirkpatrick T.R. (1989). Ergodic Behaviour in Supercooled Liquids and Glasses. Phys. Rev. A.

[25] Schön J.C., Schreuer J. (1998). Structure Prediction and Modelling of Solids: An Energy Landscape Point of View. Proceedings of RIGI-Workshop 1998.

[26] Jackson J.D. (1975). Classical Electrodynamics.

[27] Lebon G., Jou D., Casas-Vazquez J. (2008). Finite-Time Thermodynamics: Economy, Ecology, and Heat Engines.

[28] Sieniutycz S., Shiner J.S. (1994). Thermodynamics of Irreversible Processes and its Relation to Chemical Engineering: Second Law Analyses and Finite Time Thermodynamics. J. Non-Equil. Thermodyn..

[29] Nulton J.D., Salamon P., Andresen B., Anmin Q. (1985). Quasistatic processes as step equilibrations. J. Chem. Phys.

[30] Schön J.C. (1996). A thermodynamic distance criterion of optimality for the calculation of free energy changes from computer simulations. J. Chem. Phys..

[31] Rozonoer L.I., Tsirlin A.M. (1983). Optimal control of thermodynamic processes. Automat. Remote Control.

[32] Andresen B., Salamon P., Berry R.S. (1977). Thermodynamics in finite time: Extremals for imperfect heat engines. J. Phys. Chem..

[33] Muschik W., Hoffmann K.H. (2006). Endoreversible Thermodynamics: A Tool for Simulating and Comparing Processes of Discrete Systems. J. Non-Equil. Thermodyn..

[34] Wagner K., Hoffmann K.H. (2015). Endoreversible modeling of a PEM fuel cell. J. Non-Equil. Thermodyn..

[35] Jansen M., Pentin I.V., Schön J.C. (2012). A universal representation of the states of chemical matter including metastable configurations in phase diagrams. Angew. Chem. Int. Ed..

[36] Schön J.C., Doll K., Jansen M. (2010). Predicting solid compounds via global exploration of the energy landscape of solids on the ab initio level without recourse to experimental information. Phys. Stat. Sol. (b).

[37] Jansen M., Doll K., Schön J.C. (2010). Addressing chemical diversity by employing the energy landscape concept. Acta. Cryst. A.

[38] Neelamraju S., Oligschleger C., Schön J.C. (2017). The threshold algorithm: Description of the methodology and new developments. J. Chem. Phys..

[39] Struik L.C.E. (1978). Physical Aging in Amorphous Polymers and Other Materials.

[40] Lundgren L., Svedlindh P., Nordblad P., Beckman O. (1983). Dynamics of the relaxation time spectrum in a CuMn spin glass. Phys. Rev. Lett..

[41] Sibani P., Hoffmann K.H. (1997). Aging and relaxation dynamics in free-energy landscapes with multiple minima. Phys. A.

[42] Kob W., Barrat J., Sciortino F., Tartaglia P. (2000). Aging in a Simple Glass Former. J. Phys. Cond. Matter.

[43] Hannemann A., Schön J.C., Jansen M., Sibani P. (2005). Non-equilibrium Dynamics in Amorphous Si_3_B_3_N_7_. J. Phys. Chem. B.

[44] Milchev A., Binder K., Heermann D.W. (1986). Fluctuations and Lack of Self-Averaging in the Kinetics of Domain Growth. Z. Phys. B Cond. Matt..

[45] Huitema H., Van der Eerden J.P. (1999). Can Monte-Carlo simulations describe dynamics? A test on Lennard-Jones systems. J. Chem. Phys..

[46] Schön J.C. (2021). Energy landscape concepts for chemical systems under extreme conditions. J. Innov. Mater. Extrem. Cond..

[47] Balian R. (1991). From Microphysics to Macrophysics: Volume 1.

[48] Schön J.C. (2004). Enthalpy landscapes of the earth alkali oxides. Z. Anorg. Allg. Chem..

[49] Stillinger F.H., Weber T.A. (1982). Hidden Structure in Liquids. Phys. Rev. A.

[50] Bengtzelius U., Götze W., Sjölander A. (1984). Dynamics of supercooled liquids and the glass transition. J. Phys. C.

[51] Buchenau U. (2003). Energy landscape—A key concept in the dynamics of liquids and glasses. J. Phys. Cond. Matter.

[52] Doliwa B., Heuer A. (2003). What Does the Potential Energy Landscape Tell Us about the Dynamics of Supercooled Liquids and Glasses?. Phys. Rev. Lett..

[53] Goldstein M. (1969). Viscous Liquids and the Glass Transition: A Potential Energy Barrier Picture. J. Chem. Phys..

[54] Heuer A. (1997). Properties of a Glass-Forming System as Derived from its Potential Energy Landscape. Phys. Rev. Lett..

[55] Schön J.C., Sibani P. (2000). Energy and entropy of metastable states in glassy systems. Europhys. Lett..

[56] Middleton T.F., Wales D. (2001). Energy Landscapes of Some Model Glass Formers. Phys. Rev. B.

[57] Sastry S. (2006). Glass-forming liquids and the glass transition: The energy landscape approach to dynamics and thermodynamics. J. Indian Inst. Sci..

[58] Raza Z., Alling B., Abrikosov I.A. (2015). Computer simulations of glasses: The potential energy landscape. J. Phys. Cond. Matter.

[59] Muthukumar M. (1991). Entropic barrier model for polymer diffusion in concentrated polymer solutions and random media. J. Non-Cryst. Solids.

[60] Angell C.A. (1995). Formation of Glasses from Liquids and Biopolymers. Science.

[61] Angell C.A. (1997). Landscapes with Megabasins: Polyamorphism in Liquids and Biopolymers and the Role of Nucleation in Folding and Folding Diseases. Phys. D Nonlinear Phenom..

[62] Schön J.C. (2002). Energy Landscape of two-dimensional Lattice Polymers. J. Phys. Chem. A.

[63] Binder K., Baschnagel J., Paul W. (2003). Glass Transition of Polymer Melts: Test of Theoretical Concepts by Computer Simulation. Prog. Poly. Sci..

[64] Muthukumar M. (2005). Modeling polymer crystallization. Adv. Polym. Sci..

[65] Alexa P., Oligschleger C., Gröger P., Morchutt C., Vyas B., Lotsch B.V., Schön J.C., Gutzler R., Kern K. (2019). Short-range structural correlations in amorphous 2D polymers. ChemPhysChem.

[66] Tsuji Y., Prasad D.V.K., Elatresh S.F., Hoffmann R., Ashcroft N.W. (2016). Structural Diversity and Electron Confinement in Li_4_N: Potential for 0-D, 2-D, and 3-D Electrides. J. Amer. Chem. Soc..

[67] Niblett S.P., Biedermann M., Wales D.J., de Souza V.K. (2017). Pathways for diffusion in the potential energy landscape of the network glass former SiO_2_. J. Chem. Phys..

[68] Sherrington D., Kirkpatrick S. (1975). Solvable Model of a Spin Glass. Phys. Rev. Lett..

[69] Mezard M., Parisi G., Virasoro M.A. (1987). Spin Glass Theory and Beyond.

[70] Klotz T., Kobe S. (1994). Exact low energy landscape and relaxation phenomena in Ising spin glasses. Acta Phys. Slov..

[71] Sibani P., Schriver P. (1994). Local phase-space structure and low-temperature dynamics of short-range Ising spin glasses. Phys. Rev. B.

[72] Levinthal C. (1968). Are there pathways for protein folding?. J. Chim. Phys..

[73] Chelvanayagam G., Reich Z., Bringas R., Argos P. (1992). Prediction of Protein Folding Pathways. J. Mol. Biol..

[74] Fontana W., Stadler P.F., Bornberg-Bauer E.G., Griesmacher T., Hofacker I.L., Tacker M., Tarazona P., Weinberger E.D., Schuster P. (1993). RNA folding and combinatory landscapes. Phys. Rev. E.

[75] Bryngelson J.D., Onuchic J.N., Socci N.D., Wolynes P.G. (1995). Funnels, Pathways, and the Energy Landscape of Protein Folding: A Synthesis. Proteins Struct. Funct. Gen..

[76] Dill K.A., Bromberg S., Yue K., Fiebig K.M., Yee D.P., Thomas P.D., Chan H.S. (1995). Principles of protein folding—A perspective from simple exact models. Prot. Sci..

[77] Onuchic J.N., Luthey-Schulten Z., Wolynes P.G. (1997). Theory of protein folding: The energy landscape perspective. Ann. Rev. Phys. Chem..

[78] Dobson C.M., Sali A., Karplus M. (1998). Protein folding: A perspective from theory and experiment. Angew. Chem. Int. Ed. Engl..

[79] Honig B. (1999). Protein Folding: From the Levinthal Paradox to Structure Prediction. J. Mol. Biol..

[80] Hodge I.M. (1995). Physical Aging in Polymer Glasses. Science.

[81] Li M.S., Nordblad P., Kawamura H. (2001). Aging Effects in Ceramic Superconductors. Phys. Rev. Lett..

[82] Gutzow I., Schmelzer J. (1995). The Vitreous State.

[83] Sibani P., Schön J.C., Salamon P., Andersson J.O. (1993). Emergent hierarchies in complex systems. Europhys. Lett..

[84] Schön J.C., Sibani P. (1998). Properties of the energy landscape of network models for covalent glasses. J. Phys. A Math. Gen..

[85] Schön J.C. (1997). Preferential trapping on energy landscapes in regions containing deep-lying minima - the reason for the success of simulated annealing?. J. Phys. A Math. Gen..

[86] Hoffmann K.H., Schön J.C. (2005). Kinetic Features of Preferential Trapping on Energy Landscapes. Found. Phys. Lett..

[87] Bryngelson J.D., Wolynes P.G. (1987). Spin glasses and the statistical mechanics of protein folding. Proc. Nat. Acad. Sci. USA.

[88] Pannetier J., Bassas-Alsina J., Rodriguez-Carvajal J., Caignaert V. (1990). Prediction of crystal structures from crystal chemistry rules by simulated annealing. Nature.

[89] Freeman C.M., Newsam J.M., Levine S.M., Catlow C.R.A. (1993). Inorganic Crystal Structure Prediction Using Simplified Potentials and Experimental Unit Cells—Application to the Polymorphs of Titanium-Dioxide. J. Mater. Chem..

[90] Schön J.C., Jansen M. (1994). Determination of Candidate Structures for Lennard-Jones-Crystals through Cell Optimisation. Ber. Bunsenges..

[91] Bush T.S., Catlow C.R.A., Battle P.D. (1995). Evolutionary programming technique for predicting inorganic crystal structures. J. Mater. Chem..

[92] Schön J.C., Jansen M. (1995). Determination of Candidate Structures for Simple Ionic Compounds through Cell Optimisation. Comp. Mater. Sci..

[93] Takada A., Catlow C.R.A., Price G.D. (1995). Computer modelling of B_2_O_3_: Part I. New interatomic potentials, crystalline phases and predicted polymorphs. J. Phys. Cond. Mat..

[94] Wevers M.A.C., Schön J.C., Jansen M. (1999). Global Aspects of the Energy Landscape of Metastable Crystal Structures in Ionic Compounds. J. Phys. Cond. Matter.

[95] Woodley S.M., Battle P.D., Gale J.D., Catlow C.R.A. (1999). The prediction of inorganic crystal structures using a genetic algorithm and energy minimisation. Phys. Chem. Chem. Phys..

[96] Wevers M.A.C., Schön J.C., Jansen M. (2001). Characteristic regions on energy landscapes of complex systems. J. Phys. A Math. Gen..

[97] Oganov A.R., Glass C.W. (2006). Crystal structure prediction using ab initio evolutionary techniques: Principles and applications. J. Chem. Phys..

[98] Schön J.C., Jansen M. (2009). Prediction, determination and validation of phase diagrams via the global study of energy landscapes. Int. J. Mat. Res..

[99] Zagorac D., Schön J.C., Doll K., Jansen M. (2011). Structure prediction for PbS and ZnO at different pressures and visualization of the energy landscape. Acta Phys. Pol. A.

[100] Heard C.J., Schön J.C., Johnston R.L. (2015). Energy Landscape Exploration of Sub-Nanometre Copper-Silver Clusters. Chem. Phys. Chem..

[101] Skundric T., Zagorac D., Schön J.C., Pejic M., Matovic B. (2021). Crystal Structure Prediction of the Novel Cr_2_SiN_4_ Compound via Global Optimization, Data Mining, and the PCAE Method. Crystals.

[102] Berry R.S. (1993). Potential Surfaces and Dynamics: What Clusters Tell us. Chem. Rev..

[103] Wales D.J., Doye J.P.K. (1997). Global Optimization by Basin Hopping and the Lowest Energy Structures of Lennard-Jones Clusters Containing up to 110 Atoms. J. Phys. Chem. A.

[104] Wales D.J., Doye J.P.K., Miller M.A., Mortenson P.N., Walsh T.R., Prigogine I., Rice S.A. (2000). Energy Landscapes: From Clusters to Biomolecules. Advances in Chemical Physics, Volume 115.

[105] Johnston R.L. (2002). Atomic and Molecular Clusters.

[106] Ferrando R., Jellinek J., Johnston R.L. (2008). Nanoalloys: From theory to applications of alloy clusters and nanoparticles. Chem. Rev..

[107] Woodley S.M., Hamad S., Catlow C.R.A. (2010). Exploration of multiple energy landscapes for zirconia nanoclusters. Phys. Chem. Chem. Phys..

[108] Pacheco-Contreras R., Dessens-Felix M., Borbon-Gonzalez D.J., Paz-Borbon L.O., Johnston R.L., Schön J.C., Posada-Amarillas A. (2012). Tetrahelix conformations and transformation pathways in Pt_1_Pd_12_ clusters. J. Phys. Chem. A.

[109] Neelamraju S., Schön J.C., Doll K., Jansen M. (2012). Ab initio and empirical energy landscapes of (MgF_2_)_n_ clusters (n = 3, 4). Phys. Chem. Chem. Phys..

[110] Pacheco-Contreras R., Borbon-Gonzalez D.J., Dessens-Felix M., Paz-Borbon L.O., Johnston R.L., Schön J.C., Jansen M., Posada-Amarillas A. (2013). Determination of the energy landscape of Pd_12_Pt_1_ using a combined genetic algorithm and threshold energy method. RSC Adv..

[111] Neelamraju S., Oakley M.T., Johnston R.L. (2015). Chiral effects on helicity studied via the energy landscape of short (d, l)-alanine peptides. J. Chem. Phys..

[112] Rapacioli M., Tarrat N., Schön J.C. (2021). Exploring energy landscapes at the DFTB quantum level using the threshold algorithm: The case of the anionic metal cluster Au_20_^−^. Theor. Chem. Acc..

[113] Miller M.A., Wales D.J. (1999). Energy landscape of a model protein. J. Chem. Phys..

[114] Komatsuzaki T., Hoshino K., Matsunaga Y., Rylance G.J., Johnston R.L., Wales D.J. (2005). How many dimensions are required to approximate the potential energy landscape of a model protein. J. Chem. Phys..

[115] Vendruscolo M., Dobson C.M. (2005). Towards complete descriptions of the free-energy landscapes of proteins. Phil. Trans. Roy. Soc. A.

[116] Neelamraju S., Johnston R.L., Schön J.C. (2016). A Threshold-Minimization Scheme for Exploring the Energy Landscape of Biomolecules: Application to a Cyclic Peptide and a Disaccharide. J. Chem. Theo. Comp..

[117] Margerit W., Charpentier A., Maugis-Rabusseau C., Schön J.C., Tarrat N., Cortes J. (2023). IGLOO: An Iterative Global Exploration and Local Optimization Algorithm to Find Diverse Low-Energy Conformations of Flexible Molecules. Algorithms.

[118] Salomon G., Tarrat N., Schön J.C., Rapacioli M. (2023). Low-Energy Transformation Pathways between Naphtalene Isomers. Molecules.

[119] Naaz F., Chauhan M.S., Yadav K., Singh S., Kumar A., Prasad D.L.V.K. (2024). Structural Phase Transitions in Perovskite BaCeO_3_ with Data Mining and First Principles theoretical Calculations. J. Phys. Chem. C.

[120] Babin V., Roland C., Darden T.A., Sagui C. (2006). The free energy landscape of small peptides as obtained from metadynamics with umbrella sampling corrections. J. Chem. Phys..

[121] Wales D.J., Bogdan T.V. (2006). Potential energy and free energy landscapes. J. Phys. Chem. B.

[122] Schön J.C., Čančarević Ž.P., Hannemann A., Jansen M. (2008). Free enthalpy landscape of SrO. J. Chem. Phys..

[123] Salamon P., Wales D., Segall A., Lai Y.A., Schön J.C., Hoffmann K.H., Andresen B. (2016). Rate constants, timescales, and free energy barriers. J. Non-Equil. Thermodyn..

[124] Schön J.C., Putz H., Jansen M. (1996). Investigating the energy landscape of continuous systems—The threshold algorithm. J. Phys. Cond. Matter.

[125] Berry R.S., Kazakov V.A., Sieniutycz S., Szwast Z., Tsirlin A.M. (1999). Thermodynamic Optimization of Finite Time Processes.

[126] Kaushik S.C., Tyagi S.K., Kumar P. (2018). Finite Time Thermodynamics of Power and Refrigeration Cycles.

[127] Santoro M., Schön J.C., Jansen M. (2007). Finite-time thermodynamics and the gas-liquid phase transition. Phys. Rev. E.

[128] Schön J.C. (2009). Finite-Time Thermodynamics and the Optimal Control of Chemical Syntheses. Z. Anorg. Allg. Chem..

[129] Zettlemoyer A.C. (1969). Nucleation.

[130] Catlow C.R.A., DeLeeuw N.H., Anwar J., Davey R.J., Roberts K.J., Unwin P.R. (2007). Faraday Discussions 136: Crystal Growth and Nucleation.

[131] Anwar J., Zahn D. (2011). Uncovering molecular processes in crystal nucleation and growth by using molecular simulation. Angew. Chem. Int. Ed..

[132] Baldus H.P., Jansen M. (1997). Novel high-performance ceramics—Amorphous inorganic networks from molecular precursors. Angew. Chem. Int. Ed..

[133] Hannemann A., Schön J.C., Jansen M. (2005). Modeling the sol-gel synthesis route of amorphous Si_3_B_3_N_7_. J. Mater. Chem..

[134] Fischer D., Jansen M. (2002). Synthesis and Structure of Na_3_N. Angew. Chem. Int. Ed..

[135] Liebold-Ribeiro Y., Fischer D., Jansen M. (2008). Experimental substantiation of the “Energy Landscape Concept” for solids: Synthesis of a new modification of LiBr. Angew. Chem. Int. Ed..

[136] Schön J.C., Fischer D. (2023). Thin Films and Monolayers—Prediction, Modeling and Experiments. J. Innov. Mater. Extr. Cond..

[137] Fischer D., Andriyevsky B., Schön J.C. (2019). Systematics of the allotrope formation in elemental gallium. Mater. Res. Expr..

[138] Papon P., Leblond J., Meijer P.H.E. (2002). The Physics of Phase Transitions.

[139] Klesnil M., Lukas P. (1992). Fatigue of Metallic Materials.

[140] George E.P., Raabe D., Ritchie R.O. (2019). High-entropy alloys. Nat. Rev. Mater..

[141] Matovic B., Zagorac D., Cvijovic-Alagic I., Zagorac J., Butulija S., Ercic J., Hanzel O., Sedlak R., Lisnichuk M., Tatarko P. (2023). Fabrication and characterization of high-entropy pyrochlore ceramic. Bol. Soc. Esp. Ceram. Y Vidr..

[142] Denbigh K.G. (1958). Optimum temperature sequence in reactors. Chem. Eng. Sci..

[143] Horn F., Troltenier U. (1960). Über den optimalen Temperaturverlauf im Reaktionsrohr. Chem. Ing. Techn..

[144] Aris R. (1961). The determination of optimum operating conditions by the methods of dynamic programming. Z. Elektrochem..

[145] Mansson B., Andresen B. (1986). Optimal temperature profile for an ammonia reactor. Ind. Eng. Chem. Proc. Des. Develop..

[146] Schön J.C., Andresen B. (1996). Multiple Modes for the Operation of a Distillation Column. Industr. Engin. Chem. Res..

[147] Schaller M., Hoffmann K.H., Siragusa G., Salamon P., Andresen B. (2001). Numerically optimized performance of diabatic distillation columns. Comp. Chem. Engin..

[148] Schön J.C., Andresen B. (1996). Finite-Time Optimization of Chemical Reactions: *nA*⇌*nB*. J. Phys. Chem..

[149] Bak T.A., Salamon P., Andresen B. (2002). Optimal Behavior of Consecutive Chemical Reactions *A*⇌*B*⇌*C*. J. Phys. Chem. A.

[150] Corey E.J. (1967). General Methods for the Construction of Complex Molecules. Pure Appl. Chem..

[151] Corey E.J. (1991). The Logic of Chemical Synthesis—Multi-Step Synthesis of Complex Carbogenic Molecules. Angew. Chem. Int. Ed. Eng..

[152] Ugi I., Bauer J., Bley K., Dengler A., Dietz A., Fontain E., Gruber B., Herges R., Knauer M., Reitsam K. (1993). Computer-Assisted Solution of Chemical Problems - The Historical Development and the Present State of the Art of a New Discipline of Chemistry. Angew. Chem. Int. Ed. Eng..

[153] Li J.J. (2009). Name Reactions: A Collection of Detailed Mechanisms and Synthetic Applications.

[154] Schön J.C., Jansen M. (1996). A First Step towards Planning of Syntheses in Solid State Chemistry: Determination of Promising Structure Candidates using Global Optimization. Angew. Chem. Int. Ed. Eng..

[155] Schön J.C. (2015). On the way to a theory of solid state synthesis: Issues and open questions. Adv. Chem. Phys..

[156] Woodley S.M., Catlow C.R.A. (2008). Crystal structure prediction from first principles. Nat. Mater..

[157] Zurek E., Parrill A.L., Lipkowitz K.B. (2016). Discovering New Materials via A Priori Crystal Structure Prediction. Reviews in Computational Chemistry, Volume 29.

[158] Aykol M., Merchant A., Batzner S., Wei J.N., Cubuk E.D. (2025). Predicting emergence of crystals from amorphous precursors with deep learning potentials. Nat. Comput. Sci..

[159] Schön J.C. (2025). Enhancing synthesis prediction via machine learning. Nat. Comput. Sci..

[160] Noh M., Johnson D.C. (1996). Designed Synthesis of [TiSe_2_]_m_[NbSe_2_]_n_ Superlattices from Modulated Reactants. Angew. Chem. Int. Ed..

[161] Esters M., Alemayehu M.B., Jones Z., Nguyen N.T., Anderson M.D., Grosse C., Fischer S.F., Johnson D.C. (2015). Synthesis of Inorganic Structural Isomers By Diffusion-Constrained Self-Assembly of Designed Precursors: A Novel Type of Isomerism. Angew. Chem. Int. Ed..

[162] Schön J.C. (1996). Studying the Energy Hypersurface of Multi-Minima Systems—The Threshold and the Lid Algorithm. Ber. Bunsenges..

[163] Hoffmann K.H., Fischer A., Schön J.C., Wales D.J. (2022). Controlled Dynamics and Preferential Trapping on Energy Landscapes. In *Energy Landscapes*. Energy Landscapes of Nanoscale Systems.

[164] Hoffmann K.H., Schön J.C. (2013). Controlled dynamics on energy landscapes. Eur. Phys. J..

[165] Hoffmann K.H., Schön J.C. (2017). Combining pressure and temperature control in dynamics on energy landscapes. Eur. Phys. J. B.

[166] Wevers M.A.C., Schön J.C., Jansen M. (1998). Determination of structure candidates of simple crystalline AB_2_-systems. J. Solid State Chem..

[167] Wevers M.A.C. (1999). Energetische und Entropische Aspekte der Energielandschaften von MgF_2_, CaF_2_ und Li_x_Na_6−x_N_2_ (X = 0, 1, …, 6) Sowie ein Vergleich mit ab initio Rechnungen. Ph.D Thesis.

[168] Chen C., Ong S.P. (2022). A universal graph deep learning interatomic potential for the periodic table. Nat. Comput. Sci..

[169] Kirkwood J.G. (1935). Statistical Mechanics of Fluid Mixtures. J. Chem. Phys..

[170] Zwanzig R. (1954). High-Temperature Equation of State by a Perturbation Method. I. Nonpolar Gases. J. Chem. Phys..

[171] Watanabe M., Reinhardt W.P. (1990). Direct Dynamical Calculation of Entropy and Free Energy by Adiabatic Switching. Phys. Rev. Lett..

[172] Kirkpatrick S., Gelatt C.D., Vecchi M.P. (1983). Optimization by Simulated Annealing. Science.

[173] Holland J.H. (1975). Adaptation in Natural and Artificial Systems.

[174] Andresen B., Hoffmann K.H., Mosegaard K., Nulton J., Pedersen J.M., Salamon P. (1988). On lumped models for thermodynamic properties of simulated annealing problems. J. Phys. I.

[175] Salamon P., Sibani P., Frost R. (2002). Facts, Conjectures, and Improvements for Simulated Annealing.

[176] del Moral R., Grishin S., Walker L.R. (1999). Volcanic disturbances and ecosystem recovery. Ecosystems of Disturbed Ground.

[177] Carrillo U., Diaz-Villanueva V. (2021). Impacts of volcanic eruptions and early recovery in freshwater environments and organisms. Biol. Rev..

